# The contribution of soil extract composition and cyclic moisture dynamics to the physicochemical aging of superabsorbent polyacrylic acid and polyacrylamide hydrogels

**DOI:** 10.1038/s41598-026-53381-y

**Published:** 2026-05-22

**Authors:** Janina Neff, Christian Buchmann

**Affiliations:** Institute for Environmental Sciences, Group of Soil and Environmental Chemistry, IES Landau, RPTU University of Kaiserslautern-Landau, Landau, Germany

**Keywords:** Polyacrylic acid, Polyacrylamide, Drying-rewetting cycles, Soil extracts, Hydrogel aging, Chemistry, Engineering, Environmental sciences, Materials science

## Abstract

Polyacrylic acid (PAA) and polyacrylamide (PAM), two synthetic superabsorbent polymers (SAPs) commonly used, inter alia, in agriculture, can form three‑dimensional hydrogels that enhance soil water retention and soil structural stability. Yet, their potential aging and transformation under natural drying-rewetting dynamics and in contact with soil solutes remain unclear. In this study, we examined the effect of soil extracts from sand, loam, and clay soil in both a 72 h free swelling experiment (FSE) and in an incubation experiment (IE), where PAA and PAM hydrogels underwent ten successive drying-rewetting cycles. Samples were taken after cycles 0, 3, 5, and 10 and investigated for their swelling index (SI), water entrapment (^1^H proton nuclear magnetic resonance relaxometry), structural stability (rheometry), morphology (scanning electron microscopy), and surface chemistry (fourier transform infrared spectroscopy). In the FSE, PAA swelling in all soil extracts reduced SI, shortened T₂ relaxation, and increased solid-like characteristics, whereas PAM properties remained stable except when swollen in sand extract. During the IE, PAA exhibited progressive hydrogel network densification, further SI loss, T_2WL_ shortening, band intensity shifts, and microstructural densification, whereas PAM remained largely inert. Multivariate analysis confirmed that polymer identity and its interaction with the conducted drying-rewetting cycles and soil extract composition drove SAP aging. Overall, drying-rewetting cycles appear to induce irreversible chemo-structural alterations in anionic PAA, reducing its rehydration potential and promoting persistent, solid, plastic-like residues. In contrast, neutral PAM was more resilient under dynamic environmental conditions.

**Supplementary Information:**

The online version contains supplementary material available at 10.1038/s41598-026-53381-y.

## Introduction

Increasing water scarcity, heavy rainfall, and extreme precipitation pose major challenges for agriculture. Thus, respective soils and crops that are not native to arid regions are more severely impacted compared to plants already adapted to dry periods^1,2^. To increase irrigation efficiency and improve soil stability in the context of surface runoff or soil erosion, technical developments and the application of (synthetic) superabsorbent polymers (SAPs) are frequently investigated^3–6^. Synthetic SAPs, mainly low- to non-biodegradable polyacrylic acid (PAA) and polyacrylamide (PAM) are typically produced according to the biological model of natural, degradable biopolymers. As a key feature, they can typically absorb large quantities of water and build a three-dimensional hydrogel network in the soil interparticle space^7^. In this regard, crosslinking of polymer chains by dissolved multivalent cations typically leads to the formation of “junction zones”, which stabilizes the hydrogel network and govern its mechanical integrity, swelling, and environmental responsiveness^8^. By modulating soil water holding capacity, structural stability, permeability, and the availability of nutrients, fertilizers, and pesticides^9–11^, SAPs have gained attention in geotechnical applications, environmental engineering, and agriculture, e.g., to improve building foundations and concrete properties^12^, to remediate, stabilize, and reduce the bioavailability of toxic metals and various organic contaminants^13–15^, and to improve soil physicochemical properties and plant growth, respectively^16^. Therefore, outcome-oriented soil health frameworks, such as the EU’s Soil Monitoring Law (in force since 16 December 2025) and the UN Sustainable Development Goals (notably SDG 15.3 on land degradation neutrality), emphasizing resilient, multi-functional soils together with avoidance of degradation should be taken into account when using SAPs^17–20^. For example, PAA applied as a soil conditioner has been shown to increase the formation of more stable soil structures and larger soil aggregates, and to improve the retention and bioavailability of soil pore water (for more details, see the reviews of Zheng et al.^14^ and Buchmann et al.^21^.

From the application perspective, the performance of synthetic SAPs as soil amendments is governed by their fundamental physicochemical and chemo-structural properties. These include the degree of crosslinking between polymer chains, the density and accessibility of functional groups, and the resulting hydrogel network architecture controlling water uptake, retention, and mechanical stability within the soil pore space^9,10^. PAA contains ionizable carboxyl groups that form an anionic hydrogel whose swelling behavior strongly depends on ionic strength and the presence of multivalent cations capable of creating ionic crosslinks between adjacent polymer chains^2,22,23^. In contrast, PAM contains neutral amide groups and therefore exhibits weaker ionic interactions with surrounding soil solutes, generally resulting in a more structurally stable but less ion-responsive hydrogel network^24^. In soil, these intrinsic physicochemical characteristics directly determine key functional properties such as swelling capacity, polymer-water interactions, viscoelasticity/structural stability, and hydrogel morphology. All together they control the persistence, transformation, and soil conditioning effects of SAP hydrogels under changing environmental conditions^21^. In this regard, see the reviews of Brax et al.^9^, Buchmann et al.^21^, and Venkatachalam and Kaliappa^25^ for more detailed information.

To summarize all the previously mentioned SAP-related functions and modulations in soil, the term “gel effect” has been coined^9,10,26^, whereby the type, duration, and strength of the SAP-related gel effect strongly depends on various intrinsic SAP and soil properties. These include soil pH, texture, temperature, moisture, as well as environmental dynamics^21^.

Depending on the area of application and intended use, SAPs are applied to soil in different ways, including (a) mix application by evenly mixing SAP with soil about 0-20 cm through tillage, (b) spraying/sprinkle application by evenly spraying or spreading the SAP on the leave or soil surface, (c) coating or soaking seeds prior to planting, and (d) point/hole application by punctual, spatially limited incorporation of the SAP to the seed burial or root zone^14,27,28^. Although various fundamental mechanisms and processes of the gel effect in soil have been explored^10,12^ some of them are still unknown, including (a) how the combined effects of environmental dynamics and (b) respectively induced SAP transformation further modulate the physicochemical properties of both soil and the interparticulate hydrogel itself. In this regard, current research is increasingly focused on the aging and transformation processes of SAPs in soils, particularly on whether these materials may persist as solid, plastic-like, and non-degradable residues. Such residues could accumulate in the soil matrix, alter soil structure and water dynamics, and ultimately pose long-term risks for terrestrial ecosystems and environmental health^21^. Here, one of the research focuses is on natural drying-rewetting processes, in which the composition of the soil solution - its ionic profile, colloidal clay particles, and organic constituents - substantially influences the swelling behavior, functional properties, and fate of interparticulate SAP hydrogels in soil, i.e. even before additional interactions with the surrounding solid soil matrix. Thus, moisture-dependent changes in the concentration and availability of soil solution constituents must be considered and part of systematic studies in the context of SAP transformation and functionality in soil^2,9,10,21,22,24,26,29–34^.

Although SAP functionality in soil is known to decline over time, the combined effects of moisture dynamics and soil solution composition remain insufficiently understood, particularly regarding the formation of solid, plastic-like SAP residues^21^. Therefore, this study specifically addresses these knowledge gaps by integrating physicochemical, morphological, and relaxation-based analyses: For this, we investigated PAA and PAM, the two most common synthetic SAPs, either swollen in demineralized water (dH_2_O) or in three different soil extracts (sand, loam, and clay) and subjected to ten drying-rewetting cycles. At different drying-rewetting cycles, we quantified the swelling index (SI), water dynamics (^1^H proton nuclear magnetic resonance (NMR) relaxometry), microstructural stability (rheometry), and tracked chemical changes in terms of pH, electrical conductivity (EC), surface chemistry (attenuated total reflectance fourier transform infrared - ATR-FTIR), and morphological features (environmental scanning electron microscope - ESEM). ATR-FTIR was used to detect general shifts in characteristic polymer bands and the appearance of new or stronger peaks, providing molecular-level evidence of ionic crosslinking and condensation dynamics.

Concerning the effect of the soil extract composition, we hypothesized that the effect of cation charges on hydrogel properties is more important than the overall ion composition of the soil extract. Here, trivalent cations (especially Al^3+^) should promote stronger ionic crosslinks in the SAP hydrogels than divalent cations (e.g., Ca^2+^, Mg^2+^), promoting the formation of denser, less expandable network structures, with smaller pores, alongside shorter transverse relaxation times, reduced SI, and higher structural stability compared to dH_2_O controls. In the ATR-FTIR spectra, increased crosslinking degree will come along with systematic band shifts and intensity changes for characteristic absorption peaks of the two SAPs.

We hypothesized that SAP hydrogels swollen in dH_2_O retain their original properties up to a critical number of drying-rewetting cycles. Beyond this threshold, irreversible polymer-chain condensation was expected to cause network aging, reflected by shorter transverse relaxation times, reduced SI, and increased stability. However, these effects should be amplified in the three investigated soil extracts as function of pH and soil extract composition, promoting osmotic stresses and additional ionic crosslinking. Accordingly, ATR-FTIR of the hydrogels swollen in soil extracts should show these simplified band shift trends more prominently and at earlier cycles than in dH_2_O. Moreover, we hypothesized that crosslinks in the SAP hydrogels are more rapidly formed than SAP network expansion occurs during a drying-rewetting event, steadily increasing network densification. This should further reduce SI and transverse relaxation times and increase hydrogel viscosity. While the present study focuses on controlled laboratory conditions, identifying these fundamental physicochemical mechanisms represents a necessary first step before transferring SAP aging processes to biologically active and structurally complex field soils. Such mechanistic insights can support future soil and field studies aimed at evaluating SAP transformation under realistic environmental conditions.

## Materials and methods

Three well-characterized reference soils from the Agricultural Investigation and Research Institute (Speyer, Germany) were used. The soils differed in their texture (sand, clay and loam soil) and physicochemical properties (Table 1). From each soil, soil extracts were prepared by mixing soil and dH_2_O at 1:5 ratio, agitating for 24 h, and filtering through a 0.45 μm membrane. Soil extract will be referred to as sand, loam and clay in the following. Soil extracts were used to assess the effect on the hydrogel properties without additional interactions with solid soil constituents or bigger soil aggregates. This facilitates a focused investigation of the effects of varying cation concentrations, while ensuring comparability with previous studies and with the reference condition of drying-rewetting cycles in dH_2_O^2,22–24,30^. For the SAP swelling experiments, hydrogel-forming PAA powder (Viscosity average molar mass M_v_ = 4,000,000 g/mol) (Sigma-Aldrich, Germany; CAS 9003-01-4), and PAM granules (M_v_ = 15,000,000 g/mol) (Carl Roth, Germany, CAS 9003-05-8) of high molecular weight were investigated.

To assess the effects of soil extracts on SAP properties, we conducted a modified free swelling experiment (FSE) based on Brax et al.^9^ and Buchmann et al.^26^, in which freely swollen PAA and PAM hydrogels were prepared by allowing dry SAP powder/granules to completely swell for 72 h on a pre-wetted dialysis membrane (flat width 44 mm, molecular weight cut-off (MWCO) 14 kDa, Carl Roth, Germany), which was positioned in a liquid reservoir with the respective soil extract (or dH_2_O as control). After swelling, the respective SAP hydrogels were gently removed from the membrane and investigated for various physicochemical properties as listed below. The 72 h swelling period was selected to ensure equilibrium swelling based on previous studies with comparable SAP materials, where equilibrium conditions are typically reached within this timeframe.

For the incubation experiment (IE), after an initial free swelling of the respective SAPs in dH_2_O (C0), the respective soil extracts of the three soils were used for the subsequent drying-rewetting cycles. Thus, the SAP hydrogels were subjected to a total of ten drying-rewetting cycles of seven days each, including four days of drying at 30 °C and three days of rewetting in an excess of respective liquid (dH_2_O or soil extract) at 20 °C. The temperatures were chosen for methodological and scenario reasons: 20 °C was too slow for drying, while 30 °C allowed weekly cycles represented realistic summer topsoil conditions, and maintained active soil microbial processes. After the initial swelling, 3rd, 5th, and 10th drying-rewetting cycle (abbreviated as C0, C3, C5, and C10 in the text, and “cycle” in the figures), the respective PAA and PAM hydrogels were examined for the same physicochemical properties as in the previous swelling experiment. In total, 190 samples were prepared for both experiments with five replicates for each treatment.

**Table 1:** Selected physicochemical properties of the investigated soils and the respective 1:5 soil extracts used for the SAP swelling experiment.

Soil name		2.1	2.4	6 S
Soil type according to USDA/ DIN		Sand/sand (sS)	Loam/sandy loam (sL)	Clay/clayey loam (tL)
Bulk soil	Organic carbon [% C]	0.55 ± 0.10	1.83 ± 0.17	1.50 ± 0.13
	Nitrogen [% N]	0.06 ± 0.01	0.23 ± 0.02	0.17 ± 0.01
	pH	4.60 ± 0.10	7.50 ± 0.10	7.30 ± 0.04
	CEC [meq/100 g]	2.90 ± 0.20	17.40 ± 0.80	18.70 ± 1.20
	Density [g/cm³]	1.47 ± 0.06	1.18 ± 0.04	1.27 ± 0.03
	WHC_max_ [g/100 g]	32.50 ± 1.50	44.60 ± 2.20	41.60 ± 1.00
	Particle size distribution (PSD) according to USDA [%] < 0.002 mm	3.5 ± 0.90	23.7 ± 1.4	42.5 ± 3.0
	0.002-0.05 mm	8.4 ± 1.4	42.2 ± 1.4	35.10 ± 1.1
	0.05-2.0 mm	88.2 ± 0.6	33.1 ± 1.7	22.4 ± 2.5
Soil extracts	EC [µS/cm]	138 ± 1	424 ± 1	86 ± 1
	pH	7.0 ± 0.1	7.4 ± 0.1	8.2 ± 0.1
	Al^3+^ [mg/L]	0.40 ± 0.01	0.04 ± 0.00	0.11 ± 0.00
	Fe^3+^ [mg/L]	0.29 ± 0.04	0.23 ± 0.02	0.09 ± 0.02
	Mn^2+^ [mg/L]	1.17 ± 0.15	0.018 ± 0.001	0.12 ± 0.003
	Zn^2+^ [mg/L]	0.06 ± 0.01	0.007 ± 0.00	0.02 ± 0.00
	Ca^2+^ [mg/L]	31.15 ± 4.25	90.62 ± 5.10	47.51 ± 3.77
	Mg^2+^ [mg/L]	5.34 ± 0.13	5.87 ± 0.10	5.27 ± 0.14
	K^+^ [mg/L]	10.29 ± 0.90	2.92 ± 0.17	8.83 ± 0.96
	Na^+^ [mg/L]	2.62 ± 0.06	3.67 ± 0.05	2.63 ± 0.03

### Basic parameter

Before and after each drying-rewetting cycle, pH and EC of the soil extracts in the liquid reservoir were determined according to DIN EN ISO 11265^35^ and DIN 38404-5^36^ respectively, using a multi-parameter analyzer C863 (Consort, Belgium). Furthermore, the SI determines the ability of the SAPs to absorb water without dissolving and was calculated according to^10,37^ as the volume of the absorbed water by the swollen SAP hydrogel (in ml) divided by the weight of the dry polymer (in g).

### ^1^H-NMR relaxometry

Water entrapment was measured by ^1^H-NMR relaxometry using a Bruker Minispec MQ (Bruker, Germany) with a magnetic field strength of 0.176T, corresponding to a proton Larmor frequency of 7.5 MHz^26^. Transverse relaxation (T_2_) decay curves were acquired with an echo time (T_E_) set at 0.3 ms. The raw data were processed using MATLAB with an inverse Laplace transformation (ILT)^38^, based on the Butler, Reeds, and Dawson (BRD) algorithm^39^ to obtain the respective relaxation time distributions (RTDs). Following the method outlined by Buchmann et al.^40^, the 95th percentile of the sum of all amplitudes was employed (T_2WL_), consequently the relaxation time of 95% of the water protons within the sample was shorter than the T_2WL_^41^. Further, the peak positions (T_2peak_) within the RTDs were determined as the predominant water fraction in the sample^26^. To exclude possible (para)magnetic relaxation effects originating from the soil extracts, reference measurements were carried out as described by^42^. For more detailed information on this technique, see the review of Brax et al.^9^.

### Rheometry

Rheological measurements were conducted using an MCR 102 rheometer (Anton Paar, Germany) equipped with a cone-plate measuring geometry. For the measurements, a small amount of swollen SAP hydrogel was placed on the rheometer plate, followed by a resting period of 60 s to ensure undisturbed measurements. To assess the viscoelasticity of the hydrogels, additional amplitude sweep tests (AST) were performed at a constant frequency of 10 1/s for a total of 37 measurement points. The temperature was set to 20 °C, regulated by a Peltier unit. From the AST, shear stress and at the yield point (τ_YP_) as well as the maximum shear stress (τ_max_) for each sample were determined. The “yield point” determines the transition from elastic (solid-like) to viscous (liquid-like) behavior together with the irreversible breakdown of the inner sample microstructure (for more detailed information, see the review of Brax et al.^9^.

### ATR-FTIR

ATR- FTIR measurements of all samples were performed on the freeze-dried hydrogels using a Cary 630 ATR-FTIR spectrometer (Agilent Technologies, United States). The resulting data were analyzed using an open Specy R package^43^. As the spectra exhibited no significant background noise, no filtering was required during data processing. Striking absorbance bands at 4,000-3,200 1/cm (OH), 3,000-2,800 1/cm (CH), 1,870-1,550 1/cm (CO - carbonyl), 1,800-1,715 1/cm (COOR), 1,730-1,710 1/cm (COOH), 1,750-1,650 1/cm (COH), 1,620-1,550 1/cm (COO^−^), 1490-1150 (CH_2_), and 1,040 1/cm (CO - ether) for PAA, as well as 4,000-3,080 1/cm (NH) for PAM were qualitatively evaluated for all samples^44–49^.

### ESEM

For the visualization and identification of striking morphological features, ESEM images were exemplarily captured for the two SAP hydrogels at C0 and C10 using a Quanta 250 ESEM (FEI Company, United States) equipped with an Everhart Thornley secondary electron detector (ETD). Prior to the measurements, the hydrogels were freeze-dried and coated with a 30 nm thick gold layer using a Q150R S sputter coater (Quorom Technologies Ltd, United Kingdom). Measurements were performed under high vacuum (< 10^− 4^ Pa) with an acceleration voltage of 30 kV and an average spot size of 3.5. Different resolutions were employed (a) to visualize overall structural features of PAA and PAM swollen in dH_2_O and the different soil extracts and (b) to examine detailed network structures, including network density, crosslinking areas, and polymeric arrangements.

### Statistical analysis

Variations within the five replicates were presented as 95% confidence intervals (CI) of the arithmetic means. As variance homogeneity (Levene-test) and normal distribution (Shapiro-Wilks-test) were not fulfilled, multifactorial permuted ANOVAs were performed for the SI, T_2WL_, and T_2peak_ of the ^1^H-NMR relaxometry, as well as for τ_YP_ and τ_max_ of the rheological measurements. For this, the data was normalized based on Euclidean distance measurements and a permuted ANOVA was performed using the adonis2() command from the vegan package^50^. The respective measured parameters were used as response variables and the different soil extracts, polymer types, cycles, and their respective interactions as explanatory variables, respectively. Moreover, a scaled principal component analysis (PCA) was performed using the PCA() command from the FactoMineR package^51^. R version 4.3.1 (RStudio 2024.04.2) and Excel Office 16 were used to carry out all calculations and figures. All detailed statistical parameters, including degree of freedom, Sum of squares, R^2^, F-value and p-values are presented in Tables S1 and S2 of the supplementary information. As the experiment followed a multi-way design, the partial eta squared (ηp²) defined by Fleiss and Cohen was applied^48^. This measure isolates the effect of the investigated factor by excluding all other possible sources of variation in the design. It is calculated as the ratio of the sum of squares of the respective factor to the sum of this value and the residual sum of squares^52–55^.

## Results

### Basic parameter

**Fig. 1:**
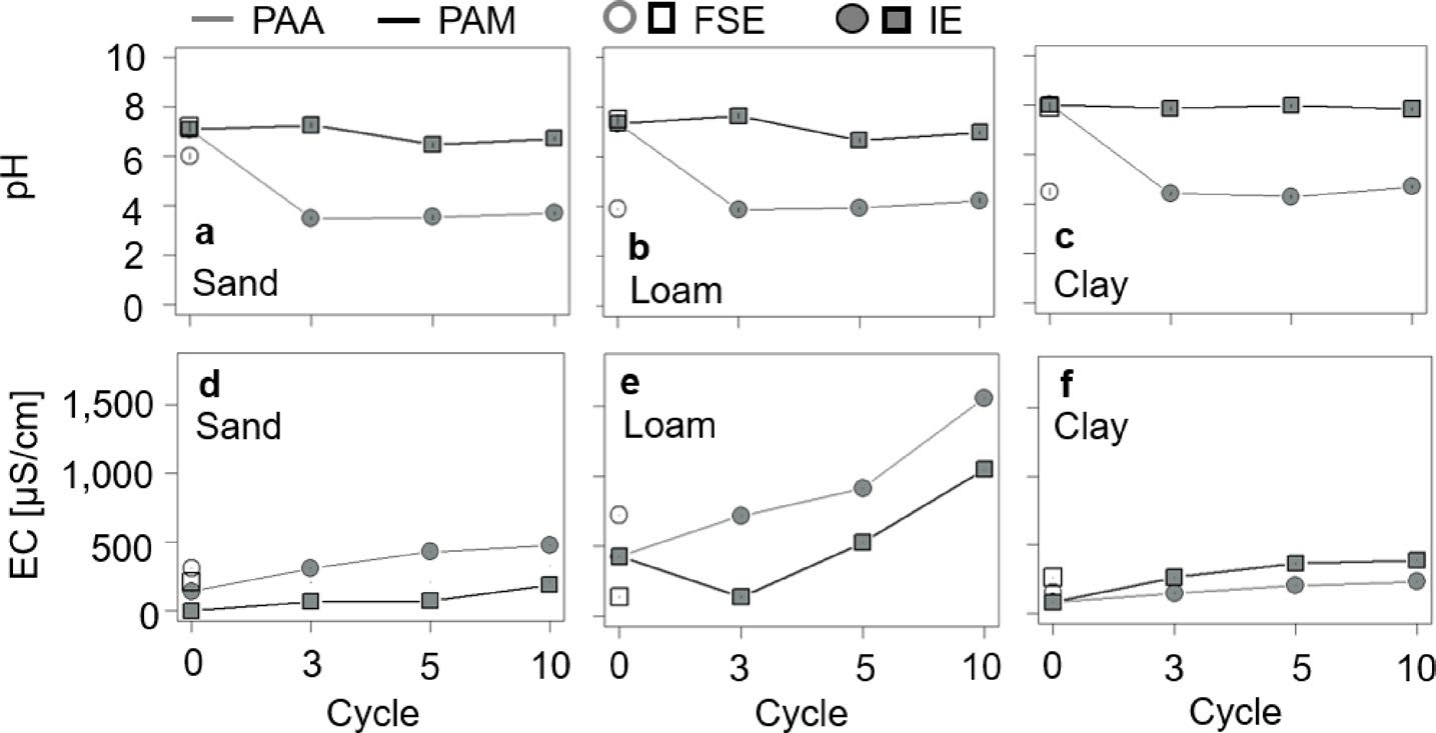
pH (**a**–**c**) and EC (**d–f**) in µS/cm in sand (**a**, **d**), loam (**b**, **e**) and clay (**c**, **f**) for both PAA and PAM-related liquid reservoirs. Open symbols represent data from the FSE, filled symbols represent data from the IE, respectively. Error bars represent the 95% CI of the arithmetic means and are not shown when smaller than the symbol size.

For the FSE, PAA substantially decreased the pH of all soil extract directly after the first swelling, from pH of 7.0 (95% CI [6.77, 7.23]), 7.4 (95% CI [7.27, 7.53]) and 8.2 (95% CI [8.04, 8.36]) to 3.6 (95% CI [3.47, 3.73]), 3.9 (95% CI [3.89, 3.91]), and 4.5 (95% CI [4.36, 4.64]) for sand, loam and clay, respectively (-49%, -47%, and − 45%, Fig. 1a-c). In contrast, PAM slightly increased the pH of the sand and loam extracts by 0.2 and 0.1 units to 7.2 (95% CI [7.01, 7.39]) and 7.5 (95% CI [7.30, 7.70]) (+ 3% and + 1%), respectively, while decreasing the pH for the clay extract by 0.3 units to 7.9 (95% CI [7.7, 8.1]) (-4%) (Fig. 1a-c). Furthermore, PAA substantially increased the EC after the first swelling in all soil extracts, from initially 138 (95% CI [137.2, 138.8]) µS/cm, 424 (95% CI [422.7, 425.3]) µS/cm, and 86 (95% CI [85.1, 86.9]) µS/cm for sand, loam and clay to 311 (95% CI [310.5, 311.5]) µS/cm, 719 (95% CI [718, 720]) µS/cm and 150 (95% CI [149.6, 150.4]) µS/cm, respectively (+ 125%, + 70%, and + 74%). Also, PAM substantially increased the EC in sand and clay extracts by 71 µS/cm and 177 µS/cm to 209 (95% CI [207.5, 210.5]) µS/cm and 263 (95% CI [262, 264]) µS/cm (+ 51% and + 206%), respectively. In contrast, PAM decreased EC in the loam extract by 286 µS/cm to 138 (95% CI [137.2, 138.8]) µS/cm (-67%) (Fig. 1d-f).

For the IE, PAA substantially decreased the pH in the respective soil extracts from C0 to C3 and remained constant for all subsequent drying-rewetting cycles. After C3, the pH of PAA-related liquid reservoirs decreased by about 51%, from 7.08 (95% CI [6.98, 7.18]), 7.34 (95% CI [7.28, 7.40]) and 8.03 (95% CI [8.00, 8.06]) to 3.5 (95% CI [3.44, 3.56]), 3.86 (95% CI [3.79, 3.93]) and 4.42 (95% CI [4.35, 5.12]) in sand, loam, and clay, respectively. In contrast, the liquid reservoirs related to PAM exhibited only minimal pH shifts (within ± 0.4 units; ± 5%) to 6.72 (95% CI [6.65, 6.79]), 6.98 (95% CI [6.91, 7.03]), and 7.84 (95% CI [7.77, 7.91]) in sand, loam, and clay until C10, respectively. EC substantially increased with drying-rewetting cycles for both SAPs, although the magnitude and pattern varied with the soil extracts (Fig. 1d-f). For PAA, EC of the liquid reservoirs steadily increased up to + 241% to + 267%, from initially 140 (95% CI [139.7, 140.3]) µS/cm, 425 (95% CI [424.4, 425.6]) µS/cm, and 81 (95% CI [80.6, 81.4]) µS/cm, (7 (95% CI [6.5, 7.5]) µS/cm for dH_2_O) to 478 (95% CI [477.8, 478.2]) µS/cm (sand), 1,558 (95% CI [1,557.95, 1,558.05]) µS/cm (loam), and 233 (95% CI [232.8, 233.2]) µS/cm (clay) after C10. In contrast, EC of the PAM-related liquid reservoir increased only modestly, by + 134%, + 146%, and + 379%, peaking at 328 (95% CI [327.3, 328.7]) µS/cm, 1,047 (95% CI [1,046.6 1,047.4]) µS/cm, and 388 (95% CI [387.8, 388.2]) µS/cm for sand, loam, and clay, respectively.

**Fig. 2:**
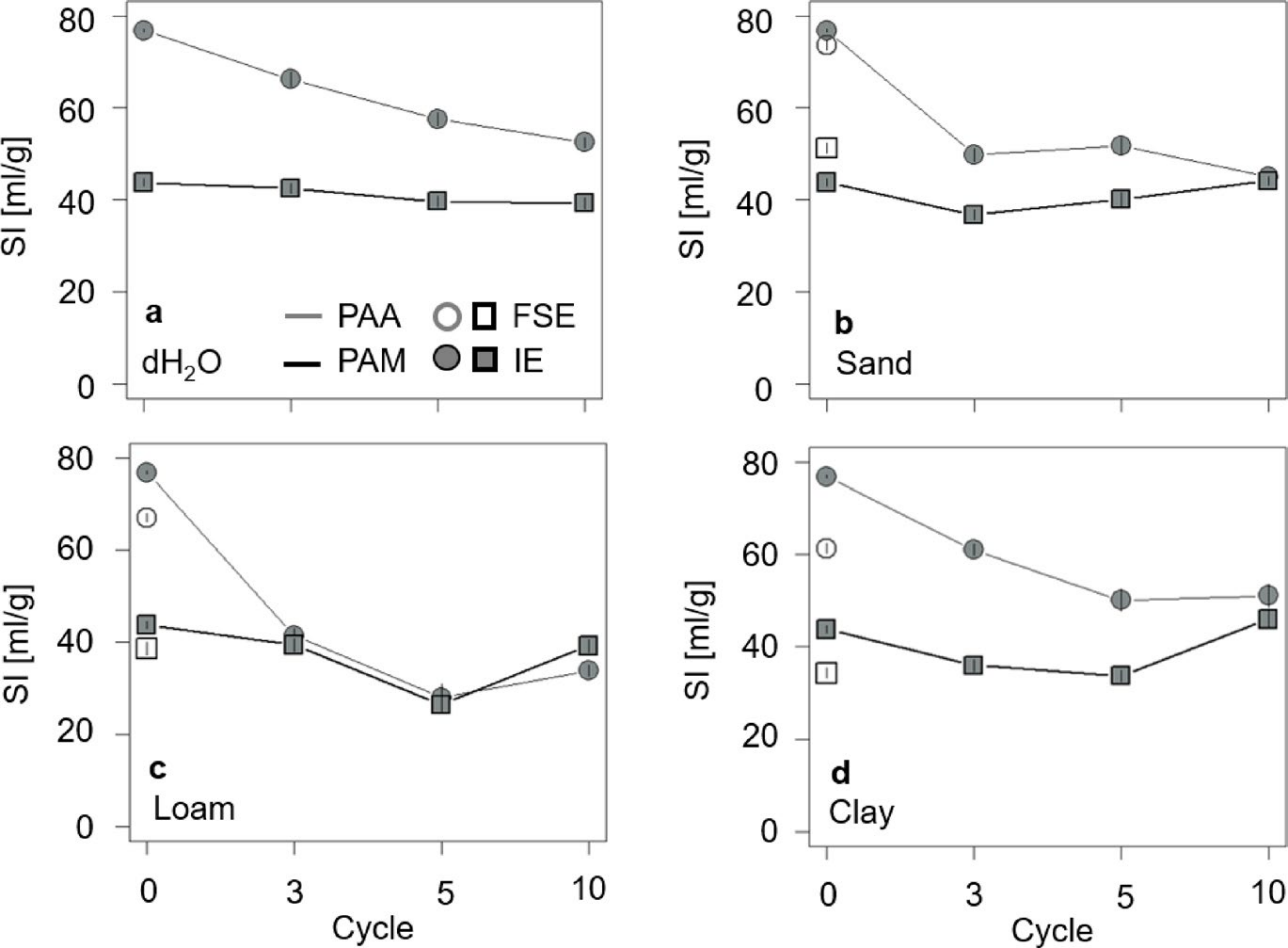
SI for both PAA (grey) and PAM (black) as function of the drying-rewetting cycles and swollen in either (**a**) dH_2_O and soil extracts of (**b**) sand, (**c**) loam, and (**d**) clay. Open symbols represent data from the FSE, filled symbols represent data from the IE, respectively. Error bars represent the 95% CI of the arithmetic means and are not shown when smaller than the symbol size.

Concerning the FSE, the SI of PAA (SI_PAA_) decreased in all three soil extracts compared to the first swelling in dH_2_O. Here, SI was the lowest for PAA swollen in clay (61.36 (95% CI [60.21, 62.51]) ml/g compared to 76.80 (95% CI [76.47, 77.13]) ml/g in dH_2_O). The SI of PAM (SI_PAM_) decreased in loam and clay, while increasing in sand to 51.23 (95% CI [50.09, 52.37]) ml/g compared to 43.80 (95% CI [43.05, 44.55]) ml/g in dH_2_O (Fig. 2). The differences in polymer type, soil extracts (including dH_2_O) and their interactions were highly significant (*p* = 0.001, Table S2). The effect sizes of the polymer type, the soil extracts and the combined effect of both showed high ɳp^2^ (0.78; 0.99; 0.5, Table S2).

In the IE, SI_PAA_ overall decreased by 20% in the course of drying-rewetting, from initially 76.80 (95% CI [76.47, 77.13]) ml/g (C0) to finally 52.5 (95% CI [51.4, 53.6]) ml/g after C10. In contrast, SI_PAM_ constantly fluctuated between 43.80 (95% CI [43.05, 44.55]) ml/g (C0) and 39.29 (95% CI [38.12, 40.46]) ml/g (after C10) throughout all drying-rewetting cycles. Concerning the effect of the soil extracts, SI_PAA_ decreased in all three soil extracts, whereby the loam showed the highest reduction of 57%, from 76.80 (95% CI [76.47, 77.13]) ml/g to 33.79 (95% CI [32.6, 34.98]) ml/g after C10. When swollen in sand, SI_PAM_ decreased about 19%, from initially 43.80 (95% CI [43.05, 44.55]) ml/g at C0 to 36.71 (95% CI [35.59, 37.83]) ml/g after C3, before (re)increasing again to 44.08 (95% CI [43.16, 45.00]) ml/g after C10. A similar pattern was observed in loam and clay: SI_PAM_ decreased to 26.44 (95% CI [24.65, 28.23]) ml/g and 33.71 (95% CI [32.56, 34.86]) ml/g, respectively, after C5, then (re)increased again to 39.22 (95% CI [37.92, 40.52]) ml/g (loam) and 45.98 (95% CI [44.07, 47.89]) ml/g (clay) after C10. Permuted ANOVAs revealed that polymer type (PAA vs. PAM), soil extract (dH_2_O, sand, loam, clay), drying-rewetting cycle (0, 3, 5, 10), and all their two- and three‐way interactions were highly significant (*p* < 0.001, Table S1) and showed effect sizes with a high effect (Fig. 2).

### Polymer-water interactions

**Fig. 3:**
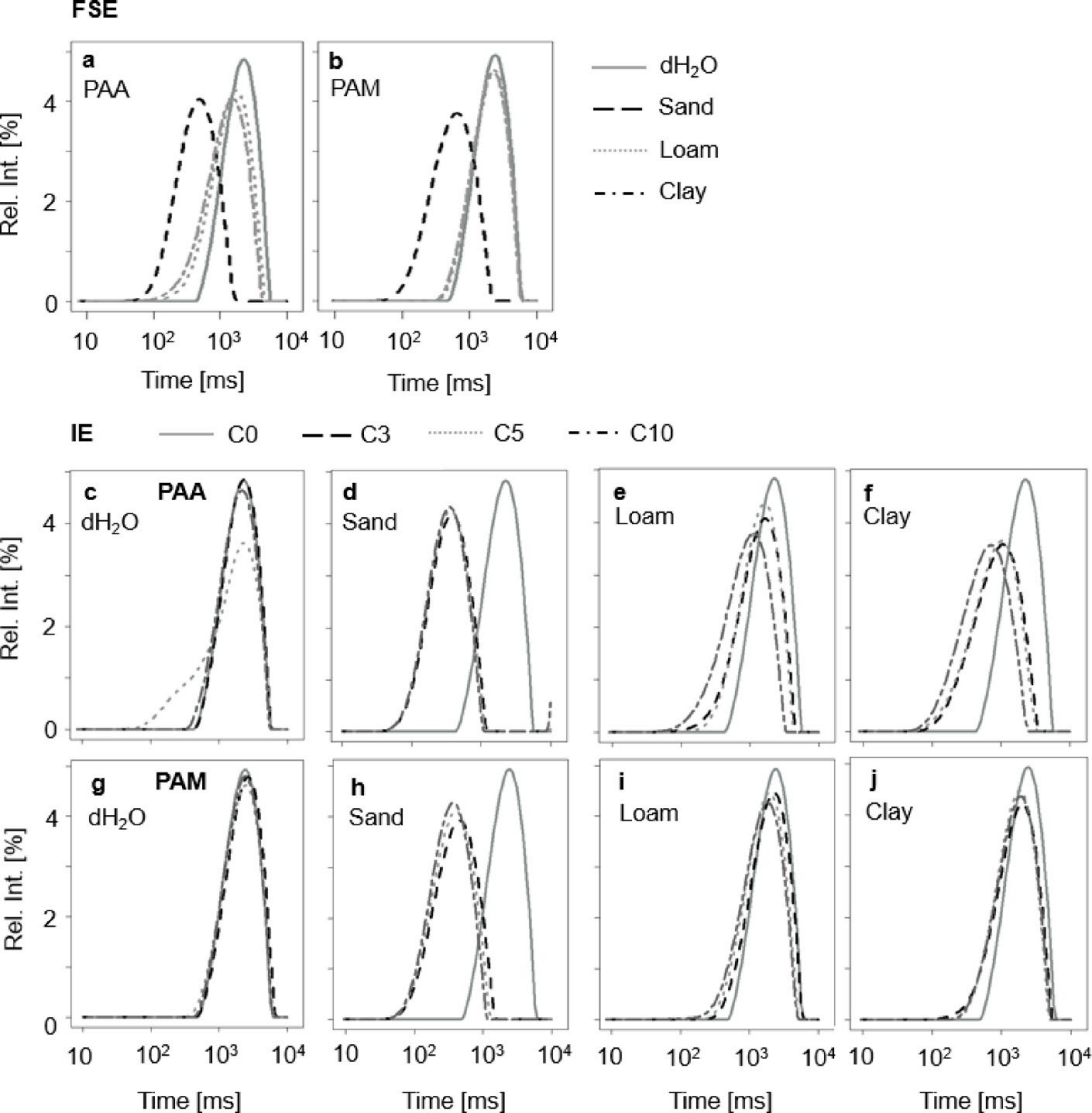
RTDs of (**a**) PAA and (**b**) PAM for the FSE in dH_2_O (grey line), and the three soil extracts - sand (black dashed line), loam (dashed line light grey) and clay (dotted-dashed line dark grey). RTD of (**c–f**) PAA and (**g**–**j**) PAM obtained from IE for the drying-rewetting cycles C0 (grey line), C3 (black dashed line), C5 (dashed line light grey) and C10 (dotted-dashed line dark grey).

**Fig. 4:**
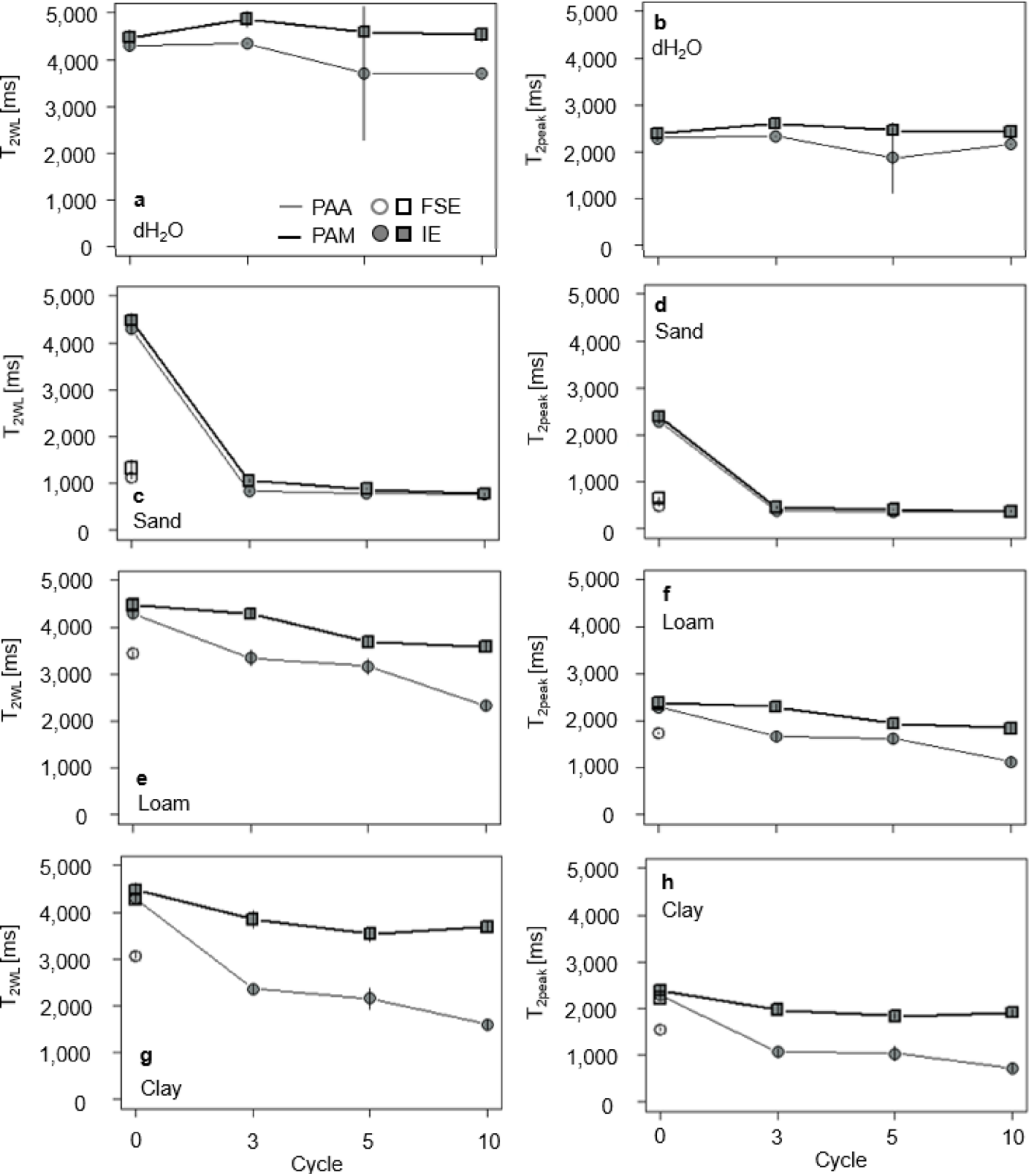
T_2WL_ (**a**, **c**, **e**, **g**) and (**b**) T_2peak_ (**b**, **d**, **f**, **h**) for PAA (grey) and PAM (black) as function of the drying-rewetting cycles for dH_2_O and soil extracts of sand, loam, and clay. Open symbols represent data from the FSE, filled symbols represent data from the IE, respectively. Error bars represent the 95% CI of the arithmetic means and are not shown when smaller than the symbol size.

The RTDs together with T_2WL_ and T_2peak_ derived from the ^1^H-NMR measurements were used to further characterize the SAP hydrogels in terms of polymer-water interactions (Figs. 3 and 4).

Concerning the FSE, both PAA and PAM exhibited the same symmetric and sharp RTDs for all three soil extracts with single T_2peak_ at 2,295 (95% CI [2,234, 2,356]) ms and 2,394 (95% CI [2,313, 2,474]) ms for dH_2_O, 1,739 (95% CI [1,692, 1,785]) ms for loam (-24%) and 1,557 (95% CI [1,479, 1.636]) ms for clay (- 32%). PAM exhibited symmetric and sharp RTDs with T_2peak_ positions at 2,471 (95% CI [2,381, 2,560]) ms for dH_2_O, 2,369 (95% CI [2,159, 2,579]) ms for loam (- 4%), and 2,233 (95% CI [2,158, 2,308]) ms for clay extracts (- 10%).

In contrast, for the first swelling in sand, both PAA and PAM exhibited as well symmetric and sharp RTDs together with decreased T_2peak_ positions to 491 (95% CI [475, 513]) ms (- 79%) for PAA and 650 (95% CI [605, 695]) ms (- 74%) for PAM (Figs. 3a and b and 4d, f and h). Regarding T_2WL_, PAA swollen in the different soil extracts showed lower values compared to dH_2_O (4,288 (95% CI [4,174, 4,402]) ms) with 1,115 (95% CI [1,097, 1,135]) ms (- 74%) for sand, 3,435 (95% CI [3,321, 3,548]) ms (- 20%) for silt, and 3,075 (95% CI [2,942, 3,207]) ms (- 28%) for clay. In contrast, PAM swollen in loam and clay showed with 4,472 (95% CI [4,322, 4,622]) ms approximately the same T_2WL_ as dH_2_O, together with a decreased T_2WL_ of 1,322 (95% CI [1,151, 1,494]) ms (- 70%) in sand (Fig. 4c, e, g).

The differences regarding the polymer type and the soil extracts including dH_2_O were highly significant (*p* = 0.001, Table S2), as well as the interaction of the polymer type with the soil extracts (*p* = 0.002). For both T_2WL_ and T_2peak_, the effect sizes showed also high influences of the polymer type (ɳp^2^ = 0.78, 0.85, Table S2), the soil extracts (ɳp^2^ = 0.97, 0.98) and their combination (ɳp^2^ = 0.49, 0.56).

For the IE, both PAA and PAM exhibited symmetric and sharp RTDs in dH_2_O with a single T_2peak_ centered at approximately 2,166 (95% CI [1,961, 2,370]) ms and 2,471 (95% CI [2,381, 2,560]) ms for all drying-rewetting cycles, respectively. However, T_2WL_ of PAA slightly decreased with each drying-rewetting cycle, from initially 4,288 (95% CI [4,174, 4,402]) ms at C0 to 3,704 (95% CI [3,704, 3,704]) ms after C5 (- 14%, Fig. 3a). Also, T_2peak_ of PAA increased from C5 on to finally 2,171 (95% CI [2,171, 2,171]) ms after C10 (+ 19%). In contrast, both T_2WL_ and T_2peak_ of PAM did not significantly shift (only by 88 ms on average), from initially 4,472 (95% CI [4,322, 4,622]) ms and 2,394 (95% CI [2,313, 2,474]) ms at C0 to 4,535 (95% CI [4,385, 4,685]) ms and 2,427 (95% CI [2,347, 2,507]) ms after C10, respectively (both + 1%).

Regarding the combined effects of soil extracts and drying-rewetting cycles, RTDs of both SAPs substantially changed, with PAM being less affected than PAA, except for sand. T_2WL_ for both PAA and PAM showed the same trend, with a decrease to 845 (95% CI [816, 873]) and 1,055 (95% CI [1,020, 1,090]) ms after C3 (- 80% and - 76%, respectively), which remained constant for all subsequent drying-rewetting cycles. Additionally, the RTD of PAA transitioned from a sharp single peak to a broader, slightly asymmetric distribution pattern.

This was also the case in loam, whereT_2WL_ of both PAA and PAM decreased with each drying-rewetting cycle to finally 2,329 (95% CI [2,229, 2,429]) ms (- 46%) and 3,580 (95% CI [3,480, 3,679]) ms (- 20%) after C10, respectively. T_2peak_ of PAA and PAM decreased accordingly to 1,625 (95% CI [1,520, 1,730]) ms (- 36%) and 1,943 (95% CI [1,878, 2,009]) ms (- 19%) after C3 and to 1,133 (95% CI [1,055, 1,211]) ms and 1,840 (95% CI [1,738, 1,943]) ms (- 23%) after C10.

For clay, T_2WL_ of PAA decreased significantly stronger (1,603 (95% CI [1,493, 1,713] ms after C10) with each drying-rewetting cycle than for the other extracts. In contrast, T_2WL_ of PAM decreased only after C3 (3,841 (95% CI [3,647, 4,035]) ms), with slight variations in the following drying-rewetting cycles. T_2peak_ showed the same course of PAA and PAM with a significantly stronger decrease of PAA from 2,295 (95% CI [2,234, 2,357]) ms at C0 to 729 (95% CI [649, 810]) ms after C10. In contrast, PAM decreased about 18%, from 2,394 (95% CI [2,313, 2,474]) ms at C0 to 1972 (95% CI [1,870, 2,075]) ms after C3 (Figs. 3c-j and 4) followed by slight variations in the following drying-rewetting cycles. The differences in polymer type, soil extract and the drying-rewetting cycles were highly significant (*p* = 0.001, Table S1). According to the p-values T_2WL_ showed high effect sizes of the polymer type (ɳp^2^ = 0.61, Table S1), the soil extracts (ɳp^2^ = 0.95), the drying-rewetting cycles (ɳp^2^ = 0.26) and the interaction of the polymer type with the soil extracts (ɳp^2^ = 0.45). Also T_2peak_ showed high effect sizes in terms of the interaction of the soil extracts and the drying-rewetting cycles (ɳp^2^ = 0.14) and the interaction of the polymer type, the soil extract and the drying-rewetting cycles (ɳp^2^ = 0.14).

### Chemo-structural properties and morphology

**Fig. 5:**
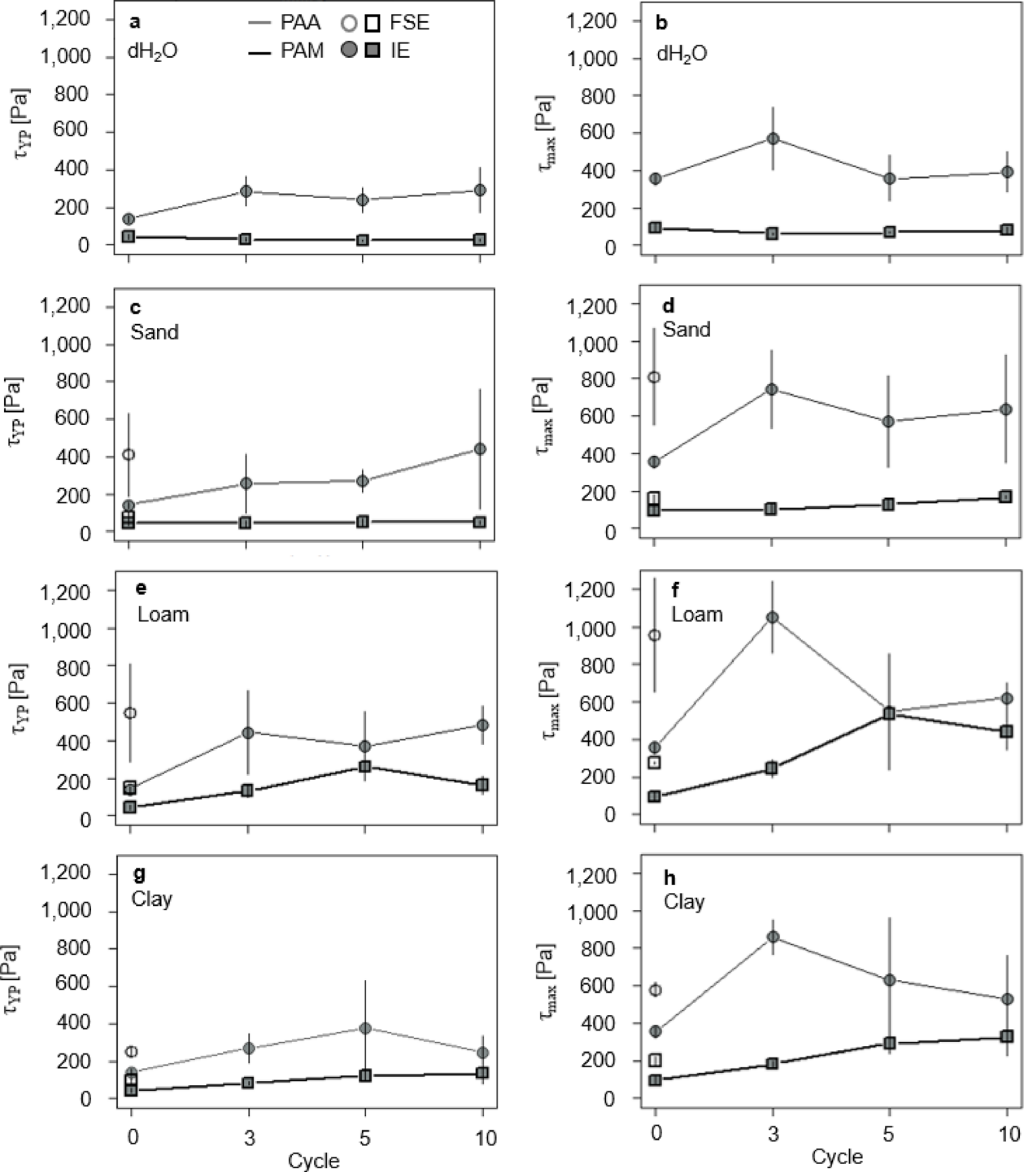
τ_YP_ (**a**, **c**, **e**, **g**) and τ_max_ (**b**, **d**, **f**, **h**) for both PAA (grey) and PAM (black) as function of the drying-rewetting cycles for (**a**, **b**) dH_2_O and soil extracts of (**c**, **d**) sand, (**e**, **f**) loam, and (**g**, **h**) clay. Open symbols represent data from the FSE, filled symbols represent data from the IE, respectively. Error bars represent the 95% CI of the arithmetic means and are not shown when smaller than the symbol size.

Concerning the FSE, both structural stability in terms of τ_YP_ and τ_max_ of PAA increased in all three soil extracts compared to the first swelling in dH_2_O (Fig. 5a-b). PAA swollen in loam revealed the highest τ_YP_ and τ_max_ of 546.78 (95% CI [287.61, 805.95]) Pa and 956.29 (95% CI [653.33, 1,259.25]) Pa (+ 287% and + 168% vs. dH_2_O), followed by 410.26 (95% CI [189.04, 631.48]) Pa and 810.19 (95% CI [553.11, 1,067.27]) Pa in sand (+ 190% and + 128%) and 248.92 (95% CI [223.66, 274.18]) Pa and 577.94 (95% CI [539.14, 616.74]) Pa in clay (+ 76% and + 62%), respectively. In contrast, τ_YP_ of PAA swollen in dH₂O was 141.37 (95% CI [123.55, 159.17]) Pa.

PAM showed the same course as PAA, but with only slight increases in τ_YP_ and τ_max_ compared to the first swelling in dH_2_O and PAA. Both τ_YP_ and τ_max_ were highest in loam (148.72 (95% CI [143.79, 153.65]) Pa and 274.62 (95% CI [266.84, 282.4]) Pa) and lowest in clay (97.75 (95% CI [86.13, 109.37]) Pa and 200.59 (95% CI [180.03, 221.15]) Pa). For both stability indices, the permuted ANOVAs revealed significances for polymer types (*p* = 0.001, Table S2), whereas only τ_max_ showed slight significance for the soil extracts including dH_2_O (*p* = 0.04). τ_YP_ showed high effect sizes of the polymer type and the soil extract with ɳp^2^ = 0.51 (Table S2) and ɳp^2^ = 0.20 while the interaction of both showed medium effect size of ɳp^2^ = 0.12.

For the IE, τ_YP_ and τ_max_ increased for both SAPs with increasing drying-rewetting cycles and independent of the soil extract. In dH_2_O, both τ_YP_ and τ_max_ of PAA increased with each drying-rewetting cycle, from initially 141.37 (95% CI [123.54, 159.17]) Pa and 356.08 (95% CI [325.3, 386.86]) Pa to 292.50 (95% CI [172.98, 412.02]) Pa (+ 107%) and 391.88 (95% CI [285.72, 498.04]) Pa (+ 10%) after C10, respectively. However, τ_YP_ of PAM remained constant at approximately 28.60 (95% CI [18.86, 38.34]) Pa over all drying-rewetting cycles, whereas τ_max_ slightly decreased by 14%, from 95.52 (95% CI [76.01, 115.03]) Pa at C0 to 82.33 (95% CI [66.11, 98.55]) Pa after C3.

For sand, τ_YP_ of PAA showed the same course as in dH_2_O by increasing about 212% from 141.37 (95% CI [123.547, 159.17]) Pa at C0 to 441.67 (95% CI [123.83, 759.51]) Pa after C10 (Fig. 5c-d). In contrast, τ_max_ increased by 79%, from 356.08 (95% CI [325.30, 386.86]) Pa at C0 to 636.95 (95% CI [349.52, 924.38]) Pa after C10 with the highest increase of 109% from C0 to 745.20 (95% CI [537.61, 952.79]) Pa after C3 followed by a 23% decrease to 573.09 (95% CI [329.88, 816.30]) Pa to after C5. Again, PAM showed a constant τ_YP_ of approximately 42.97 (95% CI [32.66, 53.28]) Pa during the drying-rewetting cycles with a slight increase of 15% to 49.41 (95% CI [32.30, 66.52]) Pa after C5. Also, τ_max_ remained approximately constant at 95.52 (95% CI [76.01, 115.03]) Pa at C0 and 103.70 (95% CI [76.59, 130.81]) Pa after C3 (+ 9%) and increased slightly to 127.46 (95% CI [100.74, 154.18]) Pa after C5 (+ 33%) and 168.07 (95% CI [161.33, 174.81]) Pa after C10 (+ 76%).

When swollen in loam, τ_YP_ of PAA increased from 141.37 (95% CI [123.54, 159.17]) Pa at C0 to finally 481.17 (95% CI [381.46, 580.88]) Pa after C10 (+ 240%), although C5 showed an intermediate decrease to 369.59 (95% CI [187.29, 551.89]) Pa (Fig. 5e-f). τ_max_ of PAA showed a similar course with a relatively higher increase, from 356.08 (95% CI [325.30, 386.86]) Pa at C0 to 621.40 (95% CI [543.46, 699.34]) Pa after C10 (+ 75%) and a peak of 1,051.80 (95% CI [864.13, 1,239.47]) after C3 (+ 195%). For PAM, τ_YP_ increased from 42.97 (95% CI [32.66, 53.28]) Pa at C0 to 261.25 (95% CI [207.95, 314.55]) Pa after C5 (+ 508%) and subsequently dropped to 162.53 (95% CI [117.43, 207.63]) Pa after C10 (+ 278%). τ_max_ showed a similar trend with an increase from 95.52 (95% CI [76.01, 115.03]) Pa at C0 to 536.17 (95% CI [485.08, 587.26]) Pa after C5 (+ 461%) and dropped to 440.06 (95% CI [345.93, 534.19]) Pa after C10 (+ 361%).

For clay, τ_YP_ of PAA increased from 141.37 (95% CI [123.54, 159.17]) Pa at C0 to 378.50 (95% CI [128.16, 628.84]) Pa after C5 (+ 168%) (Fig. 5g-h). With further drying-rewetting cycles, τ_YP_ dropped to finally 245.62 (95% CI [157.2, 334.04]) Pa after C10 (+ 74%). While τ_max_ of PAA increased to 859.71 (95% CI [768.27, 951.15]) Pa after C3 (+ 142%), it subsequently dropped to 629.49 (95% CI [296.39, 962.59]) Pa (+ 77%) and 527.32 (95% CI [295.22, 759.42]) Pa (+ 48%) after C5 and C10. In contrast, drying-rewetting cycles increased τ_YP_ of PAM from initially 42.97 (95% CI [32.66, 53.28]) Pa at C0 to 134.84 (95% CI [77.20, 192.48]) Pa after C10 (+ 214%). Also, τ_max_ showed this trend with an increase from 95.52 (95% CI [76.01, 115.03]) Pa at C0 to 327.73 (95% CI [229.78, 425.68]) Pa after C10 (+ 243%). Both τ_YP_ and τ_max_ showed high significances regarding the comparison of the different polymer types and treatments (*p* = 0.001, Table S1). Further, τ_max_ showed a highly significant interaction for the polymer types and drying-rewetting cycles (*p* = 0.001) as well. Both τ_YP_ and τ_max_ showed high effect sizes of the polymer type (ɳp^2^ = 0.49, 0.63, Table S1), the soil extracts (ɳp^2^ = 0.23, 0.40) and small effects of the drying-rewetting cycles and the interactions between the parameters. Further, τ_max_ showed high effects of the interaction of the polymer type and the cycles (ɳp^2^ = 0.25).

### ESEM

**Fig. 6:**
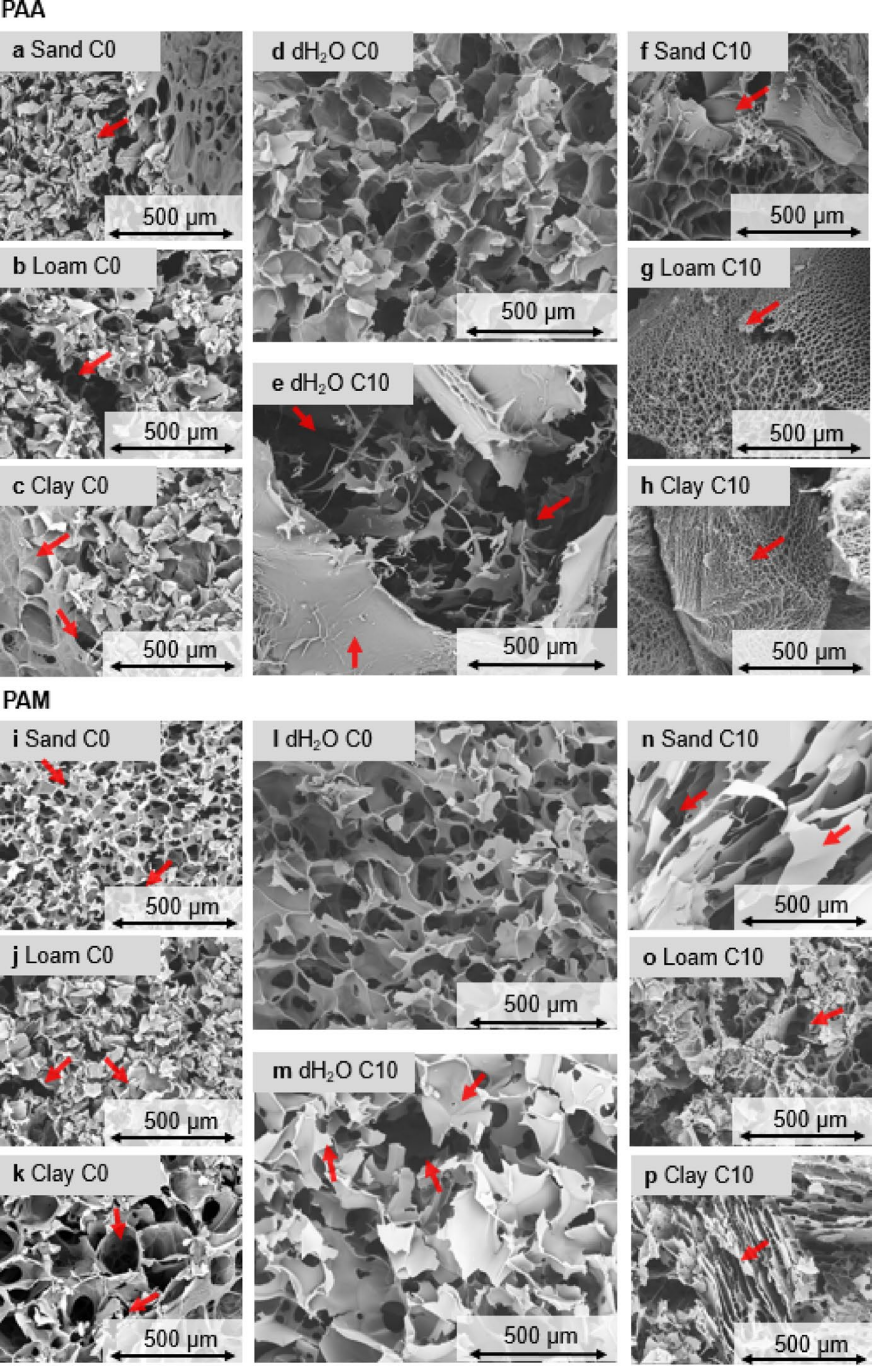
ESEM pictures of swollen and freeze-dried hydrogel networks of PAA and PAM after the first swelling (C0) in the soil extracts of sand (**a**, **i**), loam (**b**, **j**), clay (**c**, **k**), and dH_2_O (**d**, **l**) and after ten drying-rewetting cycles (C10) in dH_2_O (**e**, **m**), sand (**f**, **n**), loam (g, o) and clay (h, p). Red arrows indicate either condensed network structures, breaking edges, or porous cavities.

Concerning the FSE, ESEM images revealed morphological differences between the two SAPs, especially when swollen in sand and loam extract (Fig. 6).

Compared to dH₂O, PAA exhibited more condensed structures with fragmented, broken edges, whereas PAM formed an overall denser hydrogel network with smaller pores (arrows in figure). When swollen in clay, PAA again formed a highly condensed network with large but covered pores within layered structures. Also, PAM revealed a condensed hydrogel network with larger pores compared to the one swollen in dH_2_O.

For the IE, ESEM images revealed substantial morphological differences between the two SAPs as function of soil extracts and drying-rewetting cycles: C0 and swollen in dH_2_O caused a homogeneous network for both PAA and PAM, whereas clear differences in terms of fracture edges and condensed polymeric parts of the hydrogel network were observed after C10 for PAA.

In contrast, PAM swollen in dH_2_O still showed a relatively intact and homogenous network structure after C10, although more condensed parts and larger spaces within the network were visible. PAA swollen in sand showed highly condensed network structures after C10, together with clear breaking edges and smaller pores. It also developed thicker pore walls and a broader pore-size distribution. Also, PAM swollen in sand exhibited elongated, plate-like lamella with highly condensed areas and fan-shaped structures.

Regarding the swelling in loam and clay, the PAA hydrogel network was completely different compared to PAA at C0 and swollen in dH_2_O. However, both PAA and PAM hydrogels consisted of condensed structures and layer-like features. For loam, PAA showed irregular pore shapes with heterogeneous wall thickness and partially merged junction zones, while PAM occasionally showed granular network structures with localized microcavities and rough walls. Especially when swollen in clay, PAA formed a hydrogel network with dense, radially oriented lamellar sheets and fan-shaped domains, whereas PAM transformed into stacked, sheet-like layers with clear stratification and reduced overall pore volume.

### ATR-FTIR

**Fig. 7:**
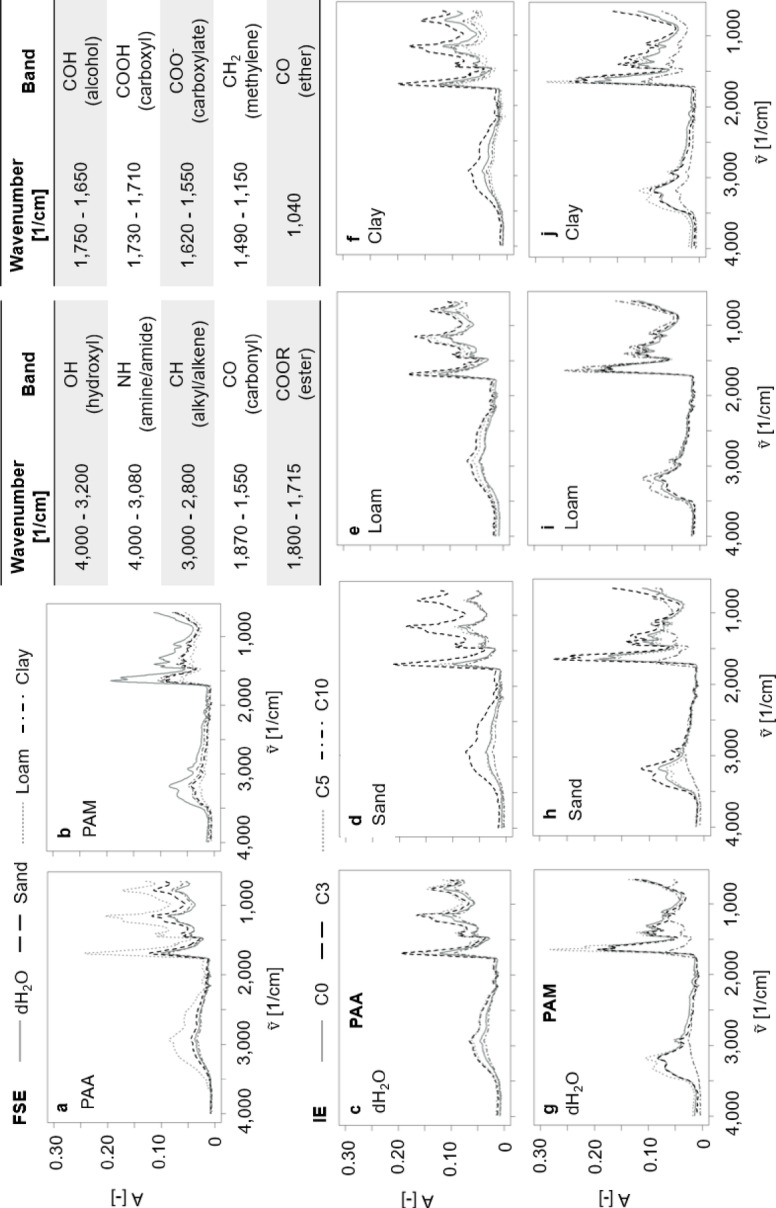
ATR-FTIR absorbance spectra of the absorbance intensity (A [-]) over the wavenumbers ($\:\stackrel{\sim}{v}$ [1/cm]) of PAA (subfigures **a**, **c–f**) and PAM (subfigures **b**, **g–j**). Subfigures a-b show the FSE with the first swelling in dH_2_O (solid grey), sand (black dashed), loam (light grey dashed) and clay (dark grey dash-dot). Subfigures c-j show the IE following drying-rewetting cycles C0 (solid grey), C3 (black dashed), C5 (light grey dashed) and C10 (dark grey dash-dot). ATR-FTIR band assignments appear in the top-right inset.

PAA initially swollen in dH_2_O water showed striking absorbance bands at 4,000-3,200 1/cm (OH), 3,000-2,800 1/cm (CH), 1870-1550 1/cm (CO), 1490-1150 (HCH), and 1,040 1/cm (CO), whereas PAM showed further absorbance bands at 3,000-3,500 1/cm (OH), 1,700-1,740 1/cm (CO), and 1,040 1/cm (CO). The complex band between 1,870 and 1,550 1/cm, hereafter referred to as the “carbonyl/carboxyl region”, comprises overlapping contributions from CO, COO^−^, and COOH, which cannot be resolved into distinct bands under the present conditions. Interestingly, both PAA and PAM also showed an obvious peak at < 1,000 1/cm (Fig. 7).

Concerning the FSE, the OH stretching and carbonyl/carboxyl stretching of PAA increased when swollen in loam but remained the same for dH_2_O, sand, and clay (Fig. 7a). Further, the CO band intensity in PAA increased markedly when swollen in loam, only slightly in sand, and remained unchanged in both dH₂O and clay. Interestingly, the band at < 1,000 1/cm showed the same course as the CO band. In contrast, PAM showed the opposite behavior as NH stretching, CO stretching and CH_2_ stretching decreased for all three soil extracts approximately the same compared to the first swelling in dH_2_O (Fig. 7b).

For the IE, drying-rewetting cycles induced cycle-dependent band intensities for PAA and all solutions: when swollen in dH_2_O, both the OH stretching (~ 3,400 1/cm) and (3,000–2,800 1/cm) CH stretching increased from C0 to reach its maximum after C3, but then decreased again after C5 and C10 (Fig. 7c). The carbonyl/carboxyl stretching (~ 1,700 1/cm), the CO band (~ 1,040 1/cm) and the < 1,000 1/cm band all followed the same pattern. In sand, the OH band again peaked after C3 before decreasing after C5 and C10 (Fig. 7d). The carbonyl/carboxyl stretching band increased from C0 to C3, decreased after C5, but (re)increased after C10. The CO band intensities showed their maxima after C3 and then decreased steadily. Interestingly, the < 1,000 1/cm band intensities showed the same course as the CO band. For loam, all four bands increased continuously with an increasing number of drying-rewetting cycles, reaching their highest intensities after C10 (Fig. 7e). When swollen in clay, each band for PAA increased sharply to peak after C3 and then decreased after C5 and C10 (Fig. 7f).

PAM showed a parallel but distinct behavior: when swollen in dH_2_O, the NH stretching band (~ 3,300 1/cm), the C-H stretching band and the CH_2_ band (~ 1,450 1/cm) increased from C0 to their maxima after C3 before decreasing again after C5 and C10 (Fig. 7g). In contrast, the amide CO band (1,650 1/cm) increased from C0 to C5 and decreased again to C10. For the sand, the intensities of the NH, CH, and CH_2_ bands again peaked after C3 and subsequently decreased, while the CO band reached its highest intensity after C5 before decreasing after C10 (Fig. 7h). PAM swollen in loam and clay resulted in the same NH, CH, and CH_2_ bands maxima after C3 and their subsequent decrease with further drying-rewetting cycles (Fig. 7i-j).

### Relationships between investigated parameters

**Fig. 8:**
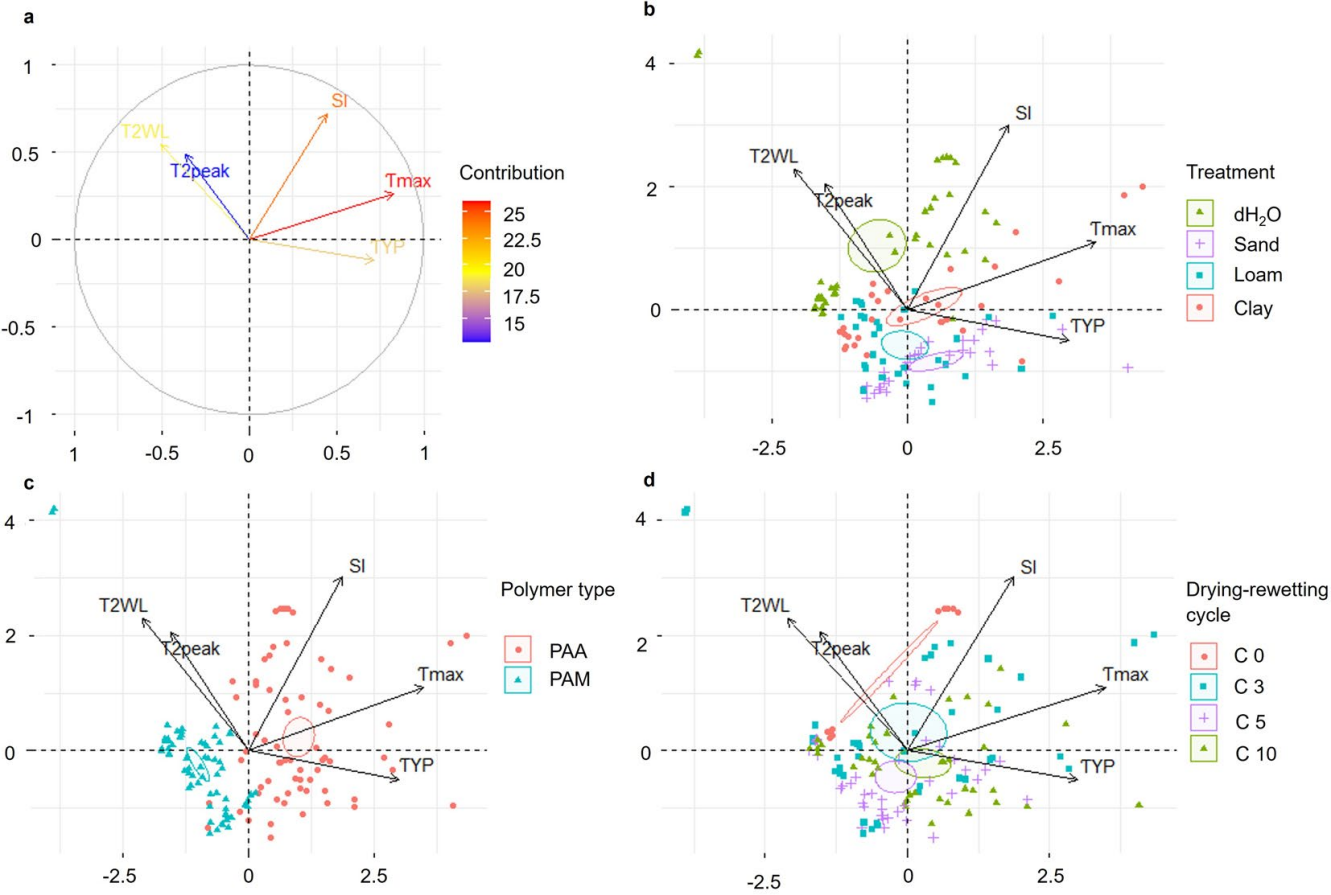
PCA of the investigated parameters showing (**a**) the contribution of the variables to PC1 and PC2 and (**b–d**) the sample clustering by the different treatments, including (**b**) the soil extracts and dH_2_O, (**c**) the different polymer types, and (**d**) the drying-rewetting cycles.

For the IE, the first two principal components accounted for 58.2% of the total variance (Dim 1 = 35.5%, Dim 2 = 22.7%) (Fig. 8). The PCA showed that SI, τ_max_, and τ_YP_ highly contributed to the explaining dimensions (Fig. 8a). Loadings indicate that SI and τ_max_ projected strongly in the positive direction of Dim1, while T_2WL_ and T_2peak_ load primarily on Dim2. τ_YP_ contributed modestly to Dim1 and slightly negatively to Dim2. In general, T_2WL_, T_2peak_ and τ_YP_ exhibited modest negative correlations, respectively. Moreover, SI and τ_max_ were also only modestly correlated.

PAA clustered predominantly on the positive side of Dim1, whereas PAM occupied the negative Dim1 region (Fig. 8b). Polymer-specific PCA revealed no relationship between T_2WL_ and T_2peak_ but did show a modest positive association between PAA and both the SI and τ_max_. These results concur with the permuted ANOVAs, which detected highly significant effects of polymer type and its interaction with drying-rewetting cycles (*p* = 0.001, ɳp^2^ = 0.86, 0.25, Table S1) and the soil extract (*p* = 0.001, ɳp^2^ = 0.93, 0.39). The three-way interaction (polymer type, drying-rewetting cycles, and soil extract) for τ_max_ was not significant (*p* = 0.08, ɳp^2^ = 0.11). The SI showed a high significance (*p* = 0.001) in terms of the polymer type itself and the interactions with soil extracts and drying-rewetting cycles.

With increasing number of drying-rewetting cycles (Fig. 8c), τ_max_ progressively shifted in the positive Dim1 direction, which was further confirmed by the permuted ANOVAs in terms of a highly significant polymer-drying-rewetting cycle interaction (*p* = 0.001, ɳp^2^ = 0.25). Moreover, SI and τ_max_ both correlated negatively with C5. SI was highly significant (*p* = 0.001) for both the drying-rewetting cycles and their interaction with polymer type and soil extract, whereas τ_max_ was only significant for the polymer type and the soil extracts (*p* = 0.001, ɳp^2^ = 0.49, 0.23).

Concerning the relevance of demineralized water and the different soil extracts, T_2WL_ and T_2peak_ projected in the positive Dim1/Dim2 quadrant alongside the hydrogels swollen in demineralized water, whereas τ_YP_ was negatively correlated. T_2peak_ showed modest significance for the interaction between soil extracts and drying-rewetting cycles (*p* = 0.011, ɳp^2^ = 0.14), and for the three-way interaction among soil extracts, polymer type, and drying-rewetting cycles (*p* = 0.018, ɳp^2^ = 0.14). T_2WL_ exhibited highly significant effects of the soil extract (*p* = 0.001, ɳp^2^ = 0.94) and its interaction with polymer type (*p* = 0.001, ɳp^2^ = 0.45), while the interactions between soil extract and drying-rewetting cycle was only modestly significant (*p* = 0.026, ɳp^2^ = 0.13).

Besides the relatively small effects of dH_2_O on τ_YP_, the effects of the polymer type and soil extracts were significant (*p* = 0.001, ɳp^2^ = 0.49, 0.23), except for the three-way interaction between soil extracts, polymer type and drying-rewetting cycles. Concerning the soil extracts, samples swollen in sand correlated negatively with T_2WL_ and T_2peak_, whereas only modestly negative correlations with SI and τ_max_ were observed when for the loam soil extracts. SI was highly significant for soil extract (*p* = 0.001, ɳp^2^ = 0.93, 0.25) and its interactions with polymer type and drying-rewetting cycles, and τ_max_ was significant for the soil extract and the polymer type alone (*p* = 0.001, ɳp^2^ = 0.39, 0.63).

## Discussion

When PAA and PAM were freely swollen in soil extracts, both SAPs hydrogels exhibited a reduced SI without significant changes in their RTDs. Compared to their initial free swelling in dH_2_O, they also showed polymer-specific increases in structural stability, further visible as more condensed hydrogel network, and uniformly decreased ATR-FTIR band intensities. This effect was more pronounced for both SAPs swollen in loam and clay. On the one hand, the observed changes in the physicochemical hydrogel properties compared to dH_2_O are related to the already well-known crosslinking of polymer chains with dissolved cations that are available in the soil extracts, typically resulting in increased hydrogel network stability as also observed in the study^23,56^. In this regard, the data also point towards a distinct role of Ca^2+^ in modifying PAA hydrogel properties. Here, the ATR-FTIR spectra consistently showed alterations in the carbonyl/carboxyl region and in the CO vibration when Ca-rich extracts (particularly loam) were present. Both bands displayed higher absorbance intensities and partial shifts to lower wavenumbers, indicating that COOH/COO^−^ groups were increasingly coordinated with Ca^2+^ and thus less available as “free” functional groups^48,49^. This was accompanied by enhanced intensities in the < 1,000 1/cm region, which was attributed to skeletal deformation modes amplified by Ca-induced hydrogel network densification. These spectral changes correspond directly to the relaxation behavior observed in the NMR measurements: PAA swollen in loam exhibited one of the strongest decreases in T_2WL_, alongside a marked shortening of T_2peak_, indicating that the increased crosslinking and hydrogel network densification restricted molecular mobility, and thus accelerating the physicochemical aging of PAA.

Concerning the soil extract composition, although sand showed a much lower Al^3+^ concentration than Ca^2+^ and Mg^2+^, it nonetheless induced the highest network densification, due to higher crosslinking, as hypothesized. By coordinating with three carboxylate groups on adjacent polymer chains, Al^3+^ forms exceptionally stable bridges^57^, driving ionotropic hydrogelation^23^, that dominates junction zone architecture. Regarding the divalent cations, Ca^2+^ still restricts (re)swelling more effectively than Mg^2+^. Consequently, the physical arrangement and morphology of the junction zones depend on cation concentration^9^. Whereby, Ca^2+^ is known to stronger reduce hydrogel (re)swelling than Mg^2+^ in terms of possible water uptake^2^, resulting in concentration- and thus crosslinking-dependent denser hydrogel networks as also shown in the ESEM images of the FSE.

On the other hand, PAA becomes deprotonated during swelling, which results in an anionic polymer hydrogel^31^. As the stability of anionic SAPs typically increases with the amount and charge of available crosslinkers in the surrounding medium^2,23,30^, high Al^3+^ and Mn^2+^/Mn^4+^ concentrations in the sand likely promoted PAA crosslinking as hypothesized. Furthermore, Amjad^58^ indicated that PAA stabilizes dissolved Mn^2+^/Mn^4+^ more efficiently than, e.g., Fe^2+^/Fe^3+^, further underlining the possible crosslinking with Mn as well. In contrast, PAM cannot be ionized due to its neutral amide group^59^, resulting in a more stable hydrogel network during swelling together with lower water absorption efficiency than PAA^24^. Consistent with this, the ATR-FTIR spectra of PAM exhibited only minor shifts in the amine/amide band and no new peak formation, indicating that network changes arise from mechanical rearrangements rather than new covalent or ionic crosslinks. The minimal ATR-FTIR shifts in PAM aligned with its unaltered RTDs and only slight rheological changes under cyclic stress, indicating an inherently stable and compact network^60^, as further underlined by the obtained ESEM images.

Besides the already mentioned crosslinking in the SAP hydrogels, the observed changes in the investigated physicochemical properties could be due to the intrinsic structure of the dry polymers themselves, as the PAM granules have a relatively low surface area compared to the investigated PAA powder, facilitating the formation of a more stable network. Here, particle-polymer interactions should be considered as e.g. clay particles could have passed the filter membranes during swelling. Especially, PAA powder promotes strong and fast interactions with soil particles like adsorption processes due to a relatively high surface area resulting in a reduced swelling potential^10,61,62^.

During the IE, PAA initially swollen in dH₂O underwent pronounced physicochemical transformations and developed increasingly solid-like behavior. This was reflected by lower water absorption, localized network densification in ESEM images, shifted RTDs in NMR, and amplified ATR-FTIR band intensities. This was in line with Yunkai et al.^24^, who reported an increased hydrogel network density due to drying-rewetting events, especially from C5 on. Also, Bai et al.^22^ reported direct relationships between the functional loss of SAPs in ultrapure water with severity of the drying events. Similar aging effects in the form of a loss of the original hydrogel properties were also observed by Gu et al.^29^ for sodium polyacrylate after 70 d of incubation in deionized solution. Consequently, the increased stability together with decreased T_2_ and shifted RTDs, reduced SI, amplified ATR-FTIR bands, and the morphological network changes observed in the ESEM images in our study support the assumption of a significant condensation of the PAA hydrogel network in the course of drying-rewetting, together with the breaking of polymer chains during network condensation and expansion. When polymer chains break and rearrange during expansion and condensation dynamics of the three-dimensional hydrogel network^29,63^, a physical rearrangement of the junction zones occurs^9^. This structural rearrangement, where polymer chains are packed more closely yet with reduced crosslinking degree, points to an increased vibrational freedom of specific functional groups (OH, NH, CO, COOH, COO^−^), enhancing dipole moment changes and thus, absorbance as shown in the ATR-FTIR spectra^64–66^.

PCA and permuted ANOVA confirmed significant interactions between soil extract composition and drying–rewetting cycles. In detail, PAA swollen in sand showed one shift towards lower RTDs for all drying-rewetting cycles together with a decrease of SI, while structural stability increased together with hydrogel network density and polymer chains breaking. This was in line with Buchmann et al.^31^, who showed that hydrogen ion release through the dissociation of the COOH group can gradually limit PAA crosslinking during its swelling together with an acidification of the surrounding solution, corresponding to the decrease in pH after PAA reswelling. Nonetheless, the authors found this effect only in sand while it disappeared in loam after one week due to its higher buffer capacity. In contrast to Buchmann et al.^31^, we recorded an uniform pH decline across all drying-rewetting cycles, which likely resulted from the use of soil extracts instead of bulk soil, which typically lacks the complex, in-situ buffering mechanisms of soil as a whole (e.g., cation exchange, OM interactions)^31,67,68^.

When subjected to drying-rewetting cycles, PAA swollen in loam and clay showed shifted RTDs with reduced T_2_, and SIs together with an increased structural stability, as also reflected in the PCA in terms of samples clustered towards the respective indices indicating increased hydrogel network densification and aging. This is consistent with the hypothesized role of multivalent cations^57^ and in line with Buchmann et al.^31^, who reported reduced PAA swelling in loam with a complete functional loss over time. Several studies have already shown, that the composition of the soil solution significantly determines both the swelling potential and the resulting physicochemical properties of PAA^10,26^. Here, ionotropic hydrogelation via cation-mediated crosslinking represents one mechanism influencing the physical arrangement of junction zones^23^. In this regard, the large and condensed areas for PAA suggested a physical rearrangement of the junction zones based on the soil extract composition^9^, and ionotropic hydrogelation dynamics^23^.

In line with these morphological changes, ATR-FTIR spectra of PAA revealed a distinct progression in functional group intensities, particularly in OH and CO bands as function of the drying-rewetting cycles. The initial increase and subsequent decline of OH absorption suggest water losses and reduced hydrogen bonding, which is consistent with the shorter T₂ relaxation times observed in the NMR data, pointing to restricted molecular mobility as the network density increases. The increase in CO signals suggests enhanced carboxylate interactions or ion-mediated crosslinking^64,69^, which is again in agreement with the results, indicating reduced water mobility, progressive network densification, and higher network stability^22,24,29,31,57^, .

Interestingly, PAA subjected to drying-rewetting cycles showed a band at < 1,000 1/cm, which increased especially when swollen in the soil extracts. In this context, the drying-rewetting-induced condensation of the hydrogel network together with the extensive crosslinking in the soil extracts may have further compacted the polymer chains, thereby amplifying skeletal deformation modes and reinforcing interactions and intensifying, e.g., out-of-plane bending and skeletal vibrations in the 900-670 1/cm region for CH and COC, respectively^47^. Again, the reduced T_2_ values reflected the more compacted hydrogel network and the soil extract-dependent amplification of crosslinking, confirming this interpretation. Further, these findings are in line with Weng et al.^70^ and Dong et al.^71^, who reported comparable spectral changes in PAA at higher crosslinking degree, arising from backbone rotations that allow short segments to adopt either helical or planar zig-zag conformations in the solid state. Taken together, the ATR-FTIR and NMR results consistently demonstrate that drying-rewetting cycles, particularly in the presence of soil extracts, accelerate hydrogel network densification, restrict molecular dynamics, and thus promote the physicochemical aging of PAA.

Contrary to PAA, PAM revealed no significantly changed hydrogel properties when swollen in dH_2_O and simultaneously subjected to drying-rewetting, as indicated by relatively stable RTDs, ATR-FTIR bands, SIs and structural stability indices throughout all drying-rewetting cycles. Although the ESEM images showed also condensed parts as for PAA, the PAM hydrogel network was still homogeneous and intact, assuming an overall lower moisture dynamics-induced effect on the physical arrangement of the junction zones^21^. These differences were further supported by the PCA results as the two SAPs clearly occupied distinct regions in the score plot, with PAA clustering in the positive direction of Dim1 - associated with higher structural rigidity and swelling loss - while PAM aligned negatively, reflecting its more stable physicochemical profile throughout the drying-rewetting cycles. These differences underscored the fundamental chemical differences between the two SAPs: when PAA hydrogel deprotonates during swelling, it results in a concentration gradient between the hydrogel network and dH_2_O. Nevertheless, subtle molecular rearrangements below the detection limits of the applied techniques cannot be entirely excluded and may require more sensitive molecular-scale analyses in future studies. However, the neutral amide group of PAM cannot be ionized^59^ and thus remains stable during swelling, causing a relatively lower water absorption efficiency compared to PAA^24^. This, in turn likely promoted the formation of an already intrinsically more stable hydrogel network with smaller pores directly after its initial swelling^60^.

Under drying-rewetting conditions, the lower structural stability, swelling pressure, and volumetric expansion potential of PAM compared to PAA further limited water uptake and reduced SI. The only minor amplification of ATR-FTIR bands and the similarity of the PAM hydrogel network to the initial swelling likely reflect inherent hydrocolloid architectural differences, particularly in junction zone formation within the three-dimensional hydrogel network^9^. Therefore, the drying-related condensation effects were relatively lower as less water was initially absorbed by the PAM hydrogel network to cause strong swelling-shrinkage dynamics and subsequent fracture formation upon drying and rewetting. Furthermore, the overall expansion and swelling pressure of the polymer chains during reswelling was further decreased by the relatively smaller pores of the PAM hydrogel and an already more stable network after the initial swelling compared to PAA^24^. This could be due to rearranged junction zones holding less water in the interstices of the hydrogel network. Moreover, PAM exhibited only minor changes in NH and CH band intensities, consistent with a chemically more stable network structure. However, the absence of pronounced spectral changes across the drying-rewetting cycles indicates lower susceptibility to hydrolysis, chain scission, or condensation reactions. This reinforces the relative resilience of PAM under dynamic moisture conditions with a preserved backbone integrity^24,60^.

Interestingly and in contrast to PAA, PAM only showed shifts in the RTDs and T_2_, whereas its structural stability remained stable over the drying-rewetting cycles and hydrogel network was only more condensed with broken polymer chains when swollen and incubated in the sand extract. This may reflect either the intrinsically higher stability of the hydrogel network following initial swelling, as previously noted, or a predominance of polymer chain-condensation over crosslinking in sand. As the neutral amide groups of PAM cannot be protonated, fewer ionic crosslinks form in sand than in loam or clay, favoring network densification over new crosslink formation. Moreover, PAM granules with their relatively lower surface area can effectively counteract both the suction tension and the confining pressure of the soil matrix^61,62^, which resulted in relatively unchanged properties of PAM compared to the initial free swelling in dH_2_O.

Besides chemical aging or changing crosslinking degree within the two hydrogels, particle-polymer interactions need to be considered, since, e.g. clay particles could have still passed the filter membranes during the drying-rewetting events and interacted with the polymer chains. Nonetheless, direct interactions with particulate soil organic matter were largely excluded in the present system as swelling experiments were conducted using filtered soil extracts. On the one hand, especially the acidic nature of PAA could have promoted interactions with clay particle surfaces and thereby mobilize exchangeable cations^32,59^. On the other hand, clay particles are generally known to limit SAP hydrogel swelling in soil by mutual swelling restriction caused during water competition^10^. However, both investigated SAPs were able to (re)swell without restrictions in our experiment, resulting in a homogeneous hydrogel network with the maximum water absorbed into the junction zones compared to their (re)swelling directly in the soil interparticle space^72–74^. Consequently, clay-induced PAA swelling restrictions were most likely lower in the soil extract than in the soil interparticle space due to (1) dilution effects since only 1:5 soil extracts were used and (2) the missing confining pressure and mutual swelling competition by the soil matrix^10^.

Although this study provides initial insights into the physicochemical aging of two commonly used SAPs as function of drying-rewetting events and soil extract composition, several limitations must be considered: first, we only investigated one specific SAP application method, namely point application, including a first swelling in dH_2_O to ensure full swelling and homogeneous hydrogel network formation without soil extract-mediated crosslinking for the first swelling. This resulted in hydrogel networks with different morphological and intrinsic properties than those directly added as dry polymers and thus swollen in the soil interparticle space^31^. Thus, soil matrix-related crosslinking, counterpressure and mutual water competition taking place directly during SAP swelling will lead to different intrinsic physicochemical hydrogel properties and should therefore be investigated further^10,26,41,61,62^.

Second, we used 1:5 soil extracts to track changes of the SAP hydrogels. Although soil extracts offer a controlled medium for assessing SAP swelling, the subsequent dilution can alter cation concentration, speciation, and pH, while excluding solid phases that typically act as adsorption sites, mechanical constraints, pore structure, and microbial or redox dynamics. Consequently, extract-based assays may overestimate SAP swelling and distort the strength and type of aging, which requires more targeted in-situ experiments^2,23,30,60^.

Third, high molecular weight polymers as used in this study are known to maximize soil-polymer interactions^75^ and increase hydrogel crosslinking degree^57^, which could have influenced the results as well. These limitations also underline the relevance of in-situ studies using native soil matrices and further commercially applied SAP products to verify the environmental relevance of the observed aging effects. Moreover, the SAP application method seems to play an important role on the density of the organ-mineral complexes^10,26,41^, as the point application used in this study has a larger surface and therefore a larger soil interface than the application way used in Buchmann et al.^31^. Future experiments should also explore varied application techniques and simulate more realistic soil moisture regimes.

Regarding the question of whether SAPs in soil can form solid, plastic-like residues^21^, our study shows that drying–rewetting cycles increased hydrogel structural stability as a function of cycle number and soil extract composition. Previous studies have shown that SAPs can form large, stable aggregates and dense organo-mineral complexes during drying, including cemented, membranous polymer structures that occupy substantial parts of the soil interparticle space^10,26,32,41^. In the present study, ATR-FTIR changes, including decreasing OH signals and shifts in carbonyl band intensities, indicate that repeated drying–rewetting induced not only physical network densification but also chemical alterations. These transformations may contribute to the formation of more persistent hydrogel residues with plastic-like characteristics. Consistently, ESEM images showed that repeated drying–rewetting cycles irreversibly densified and restructured the SAP hydrogel networks, particularly in PAA. This was associated with reduced rehydration capacity, potentially impaired long-term performance in soils, and an increased likelihood of persistent residue formation. However, although structural consolidation and reduced swelling suggest lower degradability, definitive chemical evidence of plasticization, such as novel carbon-carbon bonding motifs or enhanced resistance to microbial degradation, remains lacking. However, especially the research on targeted aging and transformation processes of SAPs is important in terms of the protection and sustainable use of agricultural soils among other areas of application like environmental engineering to remediate, stabilize toxic metals and reduce their bioavailability or in wastewater treatments^13–15,76^. To fully characterize these aging processes, future investigations should employ solid-state NMR and high-resolution mass spectrometry to resolve specific bond-level alterations^77–82^. Moreover, thermodynamic analysis in terms of the thermal stability and decomposition behaviors of the SAP hydrogels themselves and in soil appears straightforward^83–85^.

## Conclusion

This study provides important insights into the physicochemical transformation of two widely used synthetic superabsorbent polymers (SAPs), polyacrylic acid (PAA) and polyacrylamide (PAM), under repeated drying-rewetting cycles and varying soil extract compositions. SAP aging was driven by an interplay of physical densification, ion-mediated crosslinking, and chemical modifications of the hydrogel network.

In conclusion, the findings indicate that drying-rewetting cycles can cause irreversible structural and chemical changes in the two investigated SAPs as a function of soil solution composition, particularly for anionic PAA, in terms of reduced swelling capacity, increased solid-like properties with denser network structures, and altered surface chemistry. Although microbial processes were not considered in the present experimental design, previous studies generally indicate very limited microbial degradability of synthetic SAPs such as PAA and PAM in soils, underlining the relevance of possible formation of solid, plastic-like SAP residues in soil. Although ESEM, ATR-FTIR, and PCA analyses supported this interpretation in terms of changes in covalent bonds and an increased inert, less reactive solid behavior, further targeted investigations regarding long-term plasticization are required.

All in all, given the widespread application of SAPs in agriculture, environmental and geotechnical engineering, it is essential to assess and consider such processes, together with their contribution to the long-term performance, functionality, and environmental persistence of SAPs under field conditions. Future SAP design strategies aiming to increase environmental stability may therefore need to balance enhanced structural resilience with the maintenance of desired swelling functionality. Thus, future research must prioritize in-situ experiments under realistic field conditions and explore diverse SAP types (synthetic and biological), cultivation methods, and application fields, such as remediation, soil conditioning, and slope stabilization to identify SAP aging pathways and their potential relevance as environmental pollutants.

## Supplementary Information

Supplementary Information 1.

Supplementary Information 2.

## Data Availability

The data that supports the findings of this study are available from the corresponding author upon reasonable request.
