# Supplementary material for: The contribution of soil extract composition and cyclic moisture dynamics to the physicochemical aging of superabsorbent polyacrylic acid and polyacrylamide hydrogels
Source: Sci Rep. 2026 May 22;16:15983. doi: 10.1038/s41598-026-53381-y (PMC13195083; doi:10.1038/s41598-026-53381-y)
Supplement: Supplementary file 1 — Supplementary Information 1. [file 41598_2026_53381_MOESM1_ESM.pdf]

Table 1: Permutational analysis of variance (PERMANOVA) of a) T2WL, b) T2peak, c) τYP, d) τmax and e) SI as function of the polymer type, soil extract and drying-rewetting cycle (cycle) of the IE. Significant effects and interactions are shown marked in bold, respectively.

a T_{2WL}

	Df	Sum Sq	R2	F value	Pr(>F)	ηp^2
Polymer type	1	9.33	0.07	162.18	**0.001**	**0.61**
Soil extract	3	104.98	0.81	608.50	**0.001**	**0.94**
cycle	3	2.05	0.02	11.87	**0.001**	**0.26**
Polymer type: soil extract	3	4.80	0.04	27.82	**0.001**	**0.45**
Polymer type: cycle	3	0.32	0.00	1.86	0.153	0.05
Soil extract:cycle	6	0.86	0.01	2.49	**0.026**	0.13
Polymer type: soil extract:cycle	6	0.69	0.01	1.99	0.083	0.10
Residuals	104	5.98	0.05			

b T_{2peak}

	Df	Sum Sq	R2	F value	Pr(>F)	ηp^2
Polymer type	1	2.04	0.016	2.70	**0.001**	0.03
Soil extract	3	4.61	0.034	2.03	0.155	0.06
cycle	3	6.56	0.05	2.89	**0.008**	0.08
Polymer type: soil extract	3	4.52	0.04	2.00	0.081	0.05
Polymer type: cycle	3	6.55	0.05	2.89	**0.020**	0.08
Soil extract:cycle	6	13.09	0.10	2.89	**0.011**	**0.14**
Polymer type: soil extract:cycle	6	13.09	0.10	2.89	**0.018**	**0.14**
Residuals	104	78.55	0.61			

c τ_{YP}

	Df	Sum Sq	R2	F value	Pr(>F)	ηp^2
Polymer type	1	50.38	0.39	100.42	**0.001**	**0.49**
Soil extract	3	15.84	0.12	10.53	**0.001**	0.23
cycle	3	1.55	0.01	1.03	0.398	0.03
Polymer type: soil extract	3	1.11	0.01	0.74	0.525	0.02
Polymer type: cycle	3	2.01	0.02	1.34	0.276	0.04
Soil extract:cycle	6	2.10	0.02	0.70	0.646	0.04
Polymer type: soil extract:cycle	6	3.82	0.03	1.27	0.264	0.07
Residuals	104	52.18	0.40			

d τ_{max}

	Df	Sum Sq	R2	F value	Pr(>F)	ηp^2
Polymer type	1	55.59	0.43	175.35	**0.001**	**0.63**
Soil extract	3	21.50	0.02	22.61	**0.001**	**0.39**
cycle	3	2.05	0.02	2.15	0.092	0.06
Polymer type: soil extract	3	1.89	0.01	1.99	0.118	0.05
Polymer type: cycle	3	10.71	0.08	11.26	**0.001**	0.25
Soil extract:cycle	6	0.39	0.00	0.20	0.972	0.01
Polymer type: soil extract:cycle	6	3.90	0.03	2.05	0.075	0.11
Residuals	104	32.97	0.26			

e SI

	Df	Sum Sq	R2	F value	Pr(>F)	ηp^2
Polymer type	1	36.97	0.29	1271.29	**0.001**	**0.92**
Soil extract	3	41.19	0.32	472.24	**0.001**	**0.93**
cycle	3	11.71	0.09	134.27	**0.001**	**0.79**
Polymer type: soil extract	3	18.22	0.14	208.91	**0.001**	**0.86**
Polymer type: cycle	3	9.45	0.07	108.34	**0.001**	**0.76**
Soil extract:cycle	6	7.40	0.06	42.41	**0.011**	**0.71**
Polymer type: soil extract:cycle	6	1.03	0.01	5.92	**0.001**	**0.25**
Residuals	104	3.02	0.02			
