# Supplementary material for: The contribution of soil extract composition and cyclic moisture dynamics to the physicochemical aging of superabsorbent polyacrylic acid and polyacrylamide hydrogels
Source: Sci Rep. 2026 May 22;16:15983. doi: 10.1038/s41598-026-53381-y (PMC13195083; doi:10.1038/s41598-026-53381-y)
Supplement: Supplementary file 2 — Supplementary Information 2. [file 41598_2026_53381_MOESM2_ESM.pdf]

Table 2: Permutational analysis of variance (PERMANOVA) of a) T2WL, b) T2peak, c) τYP, d) τmax and e) SI as function of the polymer type and soil extract of the FSE. Significant effects and interactions are shown marked in bold, respectively.

a T_{2WL}

	Df	Sum Sq	R2	F value	Pr(>F)	ηp^2
Polymer type	1	2.66	0.09	84.61	**0.001**	0.78
Soil extract	2	24.84	0.86	394.45	**0.001**	0.97
Polymer type: soil extract	2	0.74	0.03	11.78	**0.002**	0.49
Residuals	24	0.76	0.03			

b T_{2peak}

	Df	Sum Sq	R2	F value	Pr(>F)	ηp^2
Polymer type	1	3.29	0.11	136.42	**0.001**	0.85
Soil extract	2	24.38	0.84	506.27	**0.001**	0.98
Polymer type: soil extract	2	0.75	0.03	15.64	**0.001**	0.56
Residuals	24	0.58	0.02			

c τ_{YP}

	Df	Sum Sq	R2	F value	Pr(>F)	ηp^2
Polymer type	1	12.58	0.43	25.43	**0.001**	0.51
Soil extract	2	2.97	0.10	3.00	0.058	0.20
Polymer type: soil extract	2	1.59	0.05	1.61	0.218	0.12
Residuals	24	11.87	0.41			

d τ_{max}

	Df	Sum Sq	R2	F value	Pr(>F)	ηp^2
Polymer type	1	19.26	0.66	70.08	**0.001**	0.75
Soil extract	2	2.04	0.07	3.71	**0.040**	0.24
Polymer type: soil extract	2	1.11	0.04	2.02	0.176	0.14
Residuals	24	6.59	0.23			

e SI

	Df	Sum Sq	R2	F value	Pr(>F)	ηp^2
Polymer type	1	23.43	0.81	2,377.71	**0.001**	0.99
Soil extract	2	5.09	0.18	258.37	**0.001**	0.95
Polymer type: soil extract	2	0.24	0.01	11.97	**0.001**	0.50
Residuals	24	0.24	0.01			
